# Triage of human papillomavirus infected women by methylation analysis in first-void urine

**DOI:** 10.1038/s41598-021-87329-1

**Published:** 2021-04-12

**Authors:** Severien Van Keer, Annina P. van Splunter, Jade Pattyn, Annemie De Smet, Sereina A. Herzog, Xaveer Van Ostade, Wiebren A. A. Tjalma, Margareta Ieven, Pierre Van Damme, Renske D. M. Steenbergen, Alex Vorsters

**Affiliations:** 1 Centre for the Evaluation of Vaccination (CEV), Vaccine & Infectious Disease Institute (VAXINFECTIO), Faculty of Medicine and Health Sciences, University of Antwerp, Campus Drie Eiken, Building S2, Universiteitsplein 1, 2610 Wilrijk, Belgium; 2 Amsterdam UMC, Vrije Universiteit Amsterdam, Pathology, Cancer Center Amsterdam, De Boelelaan 1117, 1081 HV Amsterdam, The Netherlands; 3 Centre for Health Economics Research and Modelling Infectious Diseases (CHERMID), Vaccine & Infectious Disease Institute (VAXINFECTIO), Faculty of Medicine and Health Sciences, University of Antwerp, Universiteitsplein 1, 2610 Wilrijk, Belgium; 4 Laboratory of Proteinscience, Proteomics & Epigenetic Signalling (PPES), Faculty of Pharmaceutical, Biomedical and Veterinary Sciences, University of Antwerp, Universiteitsplein 1, 2610 Wilrijk, Belgium; 5 Multidisciplinary Breast Clinic, Unit Gynaecologic Oncology, Department of Obstetrics and Gynaecology, Antwerp University Hospital (UZA), Wilrijkstraat 10, 2650 Edegem, Belgium; 6 Molecular Imaging, Pathology, Radiotherapy, Oncology (MIPRO), Faculty of Medicine and Health Sciences, University of Antwerp, Universiteitsplein 1, 2610 Wilrijk, Belgium; 7 Laboratory of Medical Microbiology (LMM), Vaccine & Infectious Disease Institute (VAXINFECTIO), Faculty of Medicine and Health Sciences, University of Antwerp, Universiteitsplein 1, 2610 Wilrijk, Belgium

**Keywords:** Biomarkers, Epigenetics, Cancer

## Abstract

Host cell DNA methylation analysis in urine provides promising triage markers for women diagnosed with a high-risk (HR) human papillomavirus (HPV) infection. In this study, we have investigated a panel of six host cell methylation markers (*GHSR, SST, ZIC1, ASCL1, LHX8, ST6GALNAC5*) in cervicovaginal secretions collected within the first part of the urine void (FVU) from a referral population. Cytology, histology, and HPV DNA genotyping results on paired FVU and cervical samples were available. Urinary median methylation levels from HR-HPV (n = 93) positive women were found to increase for all markers with severity of underlying disease. Significantly elevated levels were observed for *GHSR* and *LHX8* in relation to high-grade cervical intraepithelial neoplasia (CIN2 +; n = 33), with area under de curve values of 0.80 (95% Confidence Interval (CI) 0.59–0.92) and 0.76 (95% CI 0.58–0.89), respectively. These findings are the first to support the assertion that methylation analysis of host cell genes is feasible in FVU and holds promise as molecular, triage strategy to discern low- from high-grade cervical disease in HR-HPV positive women. Molecular testing on FVU may serve to increase cervical cancer screening attendance in hard-to-reach populations whilst reducing loss to follow-up and await further optimization and validation studies.

## Introduction

Cervical cancer screening based on cytology and/or (primary) detection of high-risk human papillomavirus (HR-HPV) DNA—the main etiological agent of cervical cancer—has successfully reduced cervical cancer incidence and mortality, but is hampered by suboptimal screening coverage. Though population based approaches such as sending personal invitation letters and reminders for a scheduled appointment have proven to be effective in increasing participation rates, barriers for attending cervical cancer screening have persevered^1^. These barriers can be diverse, including practical, emotional, and cognitive barriers^2^. Self-sampling could overcome part of these issues^3^ and HPV testing of self-collected cervicovaginal samples (SS) has shown similar accuracy compared to clinician-collected cervical samples (CS) using a validated PCR-based method^3,4^. Notwithstanding that SS are well-accepted by women, highest preference is given to urine self-sampling^5–10^. In recent trials, good HR-HPV DNA agreement and clinical sensitivity has been reported in first-void urine (FVU) compared to CS^6–8,11–15^. FVU allows for self-collection of cervicovaginal secretions that accumulate between the small labia and around the urethra opening, and are captured within the first part of the urine void. It is often mistaken for the first urine of the day (morning urine), which—in contrast to FVU—does not improve urinary HPV detection^6,7,16^.

Based on the same concept behind identifying more human and HPV DNA in FVU than in subsequent fractions^17–19^, FVU may also harbour other biomarkers. The use of urine to detect biomarkers for cervical screening has been receiving close appraisal in the last decade (previously reviewed^20^). Similar to the limitations of SS, FVU will likely not fulfil the high-quality cellularity standards required for morphological biomarkers such as cytology. Molecular biomarkers on the other hand have the potential to overcome this issue and are likely to yield high-throughput, objective, and reproducible results. Host cell methylation markers have shown promise as they are able to distinguish low-grade cervical intraepithelial neoplasia (CIN) with productive HR-HPV infections from high-grade CIN with transforming HR-HPV infections, especially those with a high short-term risk of progression to cancer, and detect all carcinomas^21,22^. Their clinical value to discern HR-HPV infected women with clinically relevant disease has been validated in CS^23–26^ and SS^27–30^. More recently, methylation of host cell genes in urine has shown promise as biomarker as well^31–35^. Combining primary HPV detection, and in case HR-HPV positive methylation marker triage on the same FVU sample could pose a non-invasive strategy to identify HR-HPV women with clinically relevant cervical disease in need of referral. Such strategy may be especially interesting in hard-to-reach populations where an approach based on clinician-collected samples is not effective, as well as in light of pandemics such as the current SARS-CoV-2 pandemic where access to healthcare is hampered and home-based self-sampling could aid prevention initiatives.

Therefore, the primary objective of this study was to investigate the potential of methylation analysis of six host cell genes (*GHSR, SST, ZIC1, ASCL1, LHX8,* and *ST6GALNAC5*^26,29^) in FVU as biomarker for cervical cancer prevention. These markers were previously identified by unbiased genome-wide methylation analysis in HR-HPV transformed cell lines/tissue biopsies (GHSR, SST, ZIC1)^26^ and SS (ASCL1, LHX8, and ST6GALNAC5)^29^ and validated as triage markers for HR-HPV positive women. All markers revealed a very good diagnostic performance for the detection of CIN3 + in CS and/or SS^26,29^. This study more specifically aimed to study feasibility of testing for these six methylation markers in FVU to discern underlying high-grade cervical disease from normal tissue and low-grade lesions in women diagnosed with HR-HPV.

## Materials and methods

### Study population

This cohort included 119 women (aged 25–64) referred to the Antwerp University Hospital (UZA, Belgium) colposcopy clinic due to abnormal cytology and/or infection with one or multiple HPV genotypes (January-November 2016) as described previously^11^. For methylation marker analysis, only women with a positive HR-HPV test result were included. In this subset of HR-HPV positive women, performance of each individual methylation marker was evaluated using either (i) cytology or (ii) histology outcomes as reference. The study protocol was registered on clinicialtrials.gov (NCT02714127) and approved by the central ethics committee of the University of Antwerp and UZA (B300201525585; B300201734143). All included participants signed informed consent prior to participating in study-related procedures which were in accordance with the Declaration of Helsinki (1964) and its later amendments as well as the ethical standards of the institutional review board.

### Sample collection and storage

Women collected a FVU sample with a first-void urination device (20 ml collector vial, Colli-Pee, Novosanis, Belgium) at the hospital prior to their visit with the gynaecologist for a CS and colposcopy examination. Women were requested beforehand to not extensively wash their genitals before the visit at the clinic and to not urinate at least one hour prior to this visit. Upon addition of Urine Conservation Medium (UCM, UAntwerpen^18^) in a 1:2 UCM:FVU ratio, whole UCM-buffered FVU samples were preserved on dry ice in individual aliquots within a median time span of 12 min (interquartile range (IQR): 11–16 min) after sample collection. FVU samples were subjected to batches in random order for DNA extraction, HPV DNA genotyping, and methylation of host cell genes.

Data from CS (HPV DNA genotyping, liquid based cytology (LBC)) and colposcopy (with an optional biopsy) were retrieved from the women’s medical records. CS (Cervex-Brush, Rovers Medical Devices, The Netherlands) were transferred in 20 ml collection medium (PreservCyt Solution, Hologic Europe, Belgium), analysed at UZA laboratory with the ThinPrep Pap Test (Hologic Europe), and graded according to the Bethesda classification. When indicated (according to the guidelines of the European Federation of Colposcopy), a biopsy for histological confirmation was taken during colposcopy by the clinician and graded at the UZA pathology lab using the CIN classification system. Women graded with different colposcopy and histological outcomes were classified according to the most severe stage.

### DNA extraction of first-void urine samples

DNA extraction was performed per in-house protocol developed by Vorsters A et al.^18^ as previously described^11,18^. In brief, 4 ml of UCM-buffered whole FVU was transferred to an Amicon Ultra-4 50K filter (Merck Millipore, Belgium), centrifuged (20 min; 3820 × g; 20 °C), and incubated (10 min, room temperature) after addition of 2 ml NucliSENS Lysis Buffer (BioMérieux, Benelux) to the retentate on the filter. After NucliSENS easyMag (BioMérieux, Benelux) DNA extraction, 35 of the 55 µl of DNA eluate was diluted with elution buffer (BioMérieux, Benelux) to a final volume of 75 µl used for HPV DNA genotyping. The remaining 20 µl DNA eluate was used for measuring methylation of host cell genes.

### HPV DNA genotyping using quantitative PCR

HPV DNA genotyping data for FVU and CS were generated by Riatol quantitative PCR HPV genotyping assay (qPCR) as previously described^11^. Briefly, this assay quantifies 13 HR-HPV genotypes: IARC Group 1 (HPV16/18/31/33/35/39/45/51/52/56/58/59) and 2A (HPV68); three possibly HR-HPV genotypes: IARC Group 2B (HPV53/66/67); and two low-risk (LR) HPV genotypes: IARC Group 3 (HPV6/11)^36,37^. β-globin was amplified to assess the DNA quality and to determine the concentration of human (h)DNA present in the sample. The 75 µl FVU DNA extracts were directly pipetted into the 96-well plate, followed by qPCR. For HPV DNA genotyping in CS, 400–800 µl of the remaining LBC specimens were subjected to automatic nucleic acid preparation, followed by qPCR.

### Methylation analysis of GHSR, SST, ZIC1, ASCL1, LHX8, and ST6GALNAC5 using quantitative methylation-specific PCR

Methylation analysis of a marker panel consisting of six host cell genes (*GHSR*, *SST*, *ZIC1*, *ASCL1*, *LHX8*, and *ST6GALNAC5*) was performed as described before^26,29,38,39^. In brief, 250 ng of isolated FVU DNA (or 20 µl when concentration was < 12.5 ng/µl) was subjected to bisulphite treatment using the EZ DNA Methylation Kit (Zymo Research, Orange, CA, USA). For multiplex qMSP, 2.5 µl of bisulphite converted DNA (≤ 50 ng) was added to 10 µl amplification mix^26,29,38,39^. The housekeeping gene *ACTB* was used to assess DNA quality and successful bisulphite conversion. Cycle threshold (CT)-values of methylation markers were normalised to the reference gene *ACTB* using the comparative method (2^−ΔCT^ × 100)^40^, obtaining methylation marker ratios. As a threshold for sample validity, a CT-value below or equal to 32 for *ACTB* was required for each sample.

### Statistical analysis

The Cohen’s Kappa (κ) was calculated to assess the HPV genotype agreement between paired samples and was judged as follows: κ ≤ 0.20, poor; 0.21 ≤ κ ≤ 0.40, fair; 0.41 ≤ κ ≤ 0.60, moderate; 0.61 ≤ κ ≤ 0.80, good; and κ ≥ 0.81, very good agreement^41^. Based on a good κ-agreement for HR-HPV DNA in paired FVU and CS, women with a positive HR-HPV DNA test result in FVU and/or CS were included for methylation marker analysis. Square root transformed methylation marker ratios (√(CT ratio)) were visualized according to cytology and histology classification via scatter plots with overlying box plots, indicating median methylation levels and according interquartile ranges (25th and 75th percentile). For each methylated gene, differences in marker ratios according to disease outcome (HSIL +, CIN2 +, or CIN3) were tested using the Mann Whitney U-test. The performance of each host cell methylation gene was visualized by a receiver operating characteristic (ROC)-curve, and evaluated by the area under the curve (AUC). The 95% CI for AUC were derived by using 5,000 bootstrap samples. Statistical analyses were performed at a significance level of 5% using the statistical software JMP Pro 13.

## Results

### Study population

**Figure 1:**
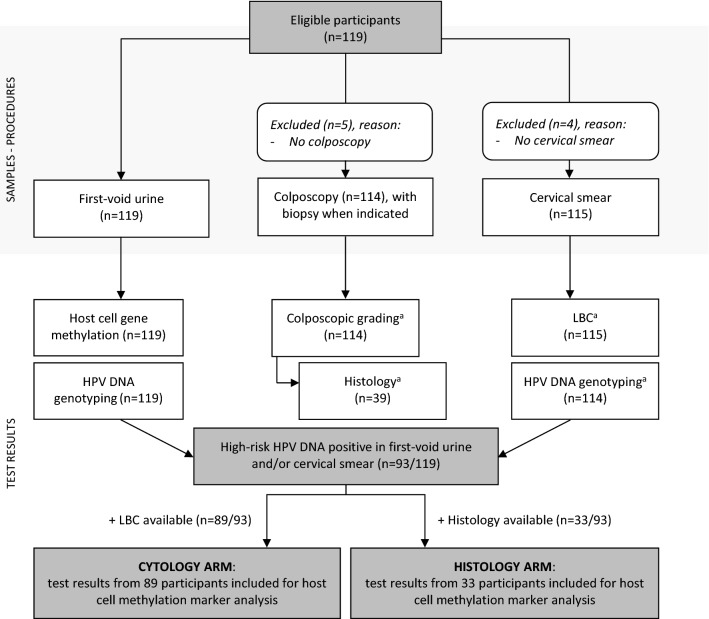
Flow diagram for the inclusion of study participants, samples, and medical records. Results from 119 participants were used for HPV DNA genotyping in first-void urine and cervical samples. Test results from high-risk HPV DNA positive women (in first-void urine and/or cervical smear, n = 93) were used to examine methylation marker performance; using either cytology results (CYTOLOGY ARM), or histology outcomes (HISTOLOGY ARM) as reference. ^a^When unavailable at D0 (day of study visit, i.e. first-void urine collection), the HPV DNA genotyping, liquid based cytology (LBC), colposcopy, and/or histology results from D0 ± 3 months were included for data analysis instead. Thus, three additional HPV DNA genotyping results were included, as well as four LBC and colposcopy, and six histology results.

The median participant age in our referral population (n = 119, Fig. 1) was 36 years (IQR: 29–44 years old). Samples from 119 (FVU) and 114 (CS) women were available for HPV DNA genotyping. All samples were valid, indicated by measurable amounts of β-globin, using qPCR^36,42^. From the 114 women with HPV genotyping results available for both FVU and CS, a good Cohen’s Kappa (κ) agreement was observed for HR-HPV (κ: 0.647; 95% confidence interval (CI): 0.494–0.800), whereas this agreement was found to be excellent for HPV16/18 (κ: 0.905; 95% CI: 0.814–0.996). Samples from 93 women with a positive test result in FVU and/or CS for HR-HPV (71 FVU + /CS +; 15 FVU + /CS−; 7 FVU−/CS +) were selected for methylation marker analysis. Performance of each methylation marker was evaluated using either (i) cytology or (ii) histology outcomes as reference, available for 89 and 33 out of the 93 HR-HPV positive samples, respectively. Demographics and HPV genotype/viral load data are detailed in Supplementary Table S1 and Van Keer et al.^11^.

### Methylation marker levels in first-void urine according to disease outcome

#### Cytology endpoints

**Figure 2:**
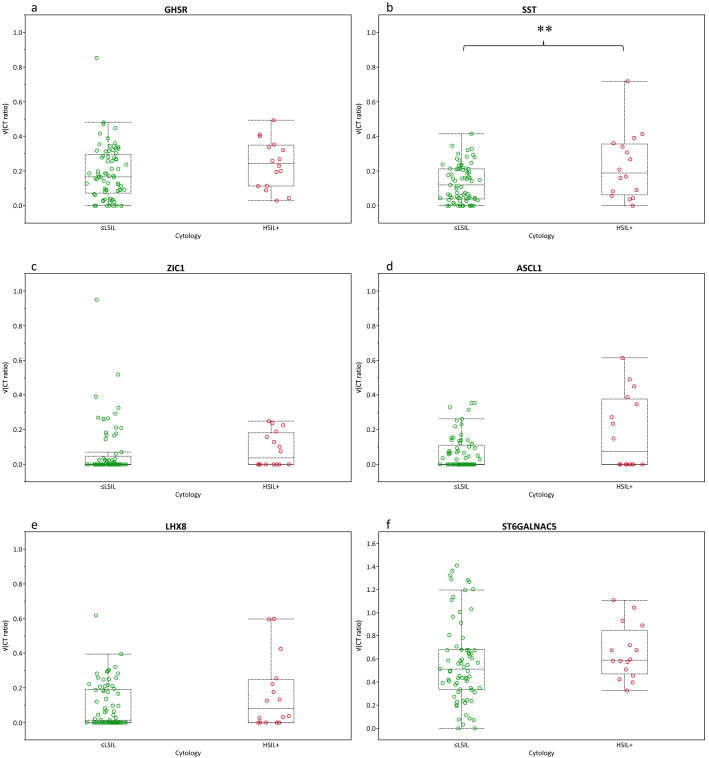
Difference in methylation ratios (square root transformed; y-axis) for normal/low-grade and high-grade cervical disease according to cytology outcome (HSIL +; x-axis); ≤ LSIL (green circles, n = 73) and HSIL + (red circles, n = 16). Box-plots indicate median methylation levels of (**a**) *GHSR*, (**b**) *SST*, (**c**) *ZIC1*, (**d**) *ASCL1*, (**e**) *LHX8*, and (**f**) *ST6GALNAC5* and according interquartile ranges (25th and 75th percentile). P-values (Mann Whitney U-test) indicated by a double asterisk mean that the null hypothesis (H0) is rejected (p ≤ 0.05) and that the median square root transformed methylation marker ratios between normal/low-grade and high-grade cervical disease are not equal. No trends were observed (0.05 < p ≤ 0.10).

Firstly, we analysed the methylation levels in relation to cytology using the complete series of HR-HPV positive samples. A set of 89 out of 93 HR-HPV positive samples with cytology endpoint available were selected (Fig. 1), resulting in 73 ≤ LSIL (NILM, ASC-US, LSIL) and 16 HSIL + (HSIL, ASC-H) cases. Increased median methylation levels were observed for all markers in HSIL + as to ≤ LSIL, with a significant increase observed for *SST* (Fig. 2). All tested FVU samples were valid for the qMSP (CT-value *ACTB* ≤ 32).

#### Histology endpoints

**Figure 3:**
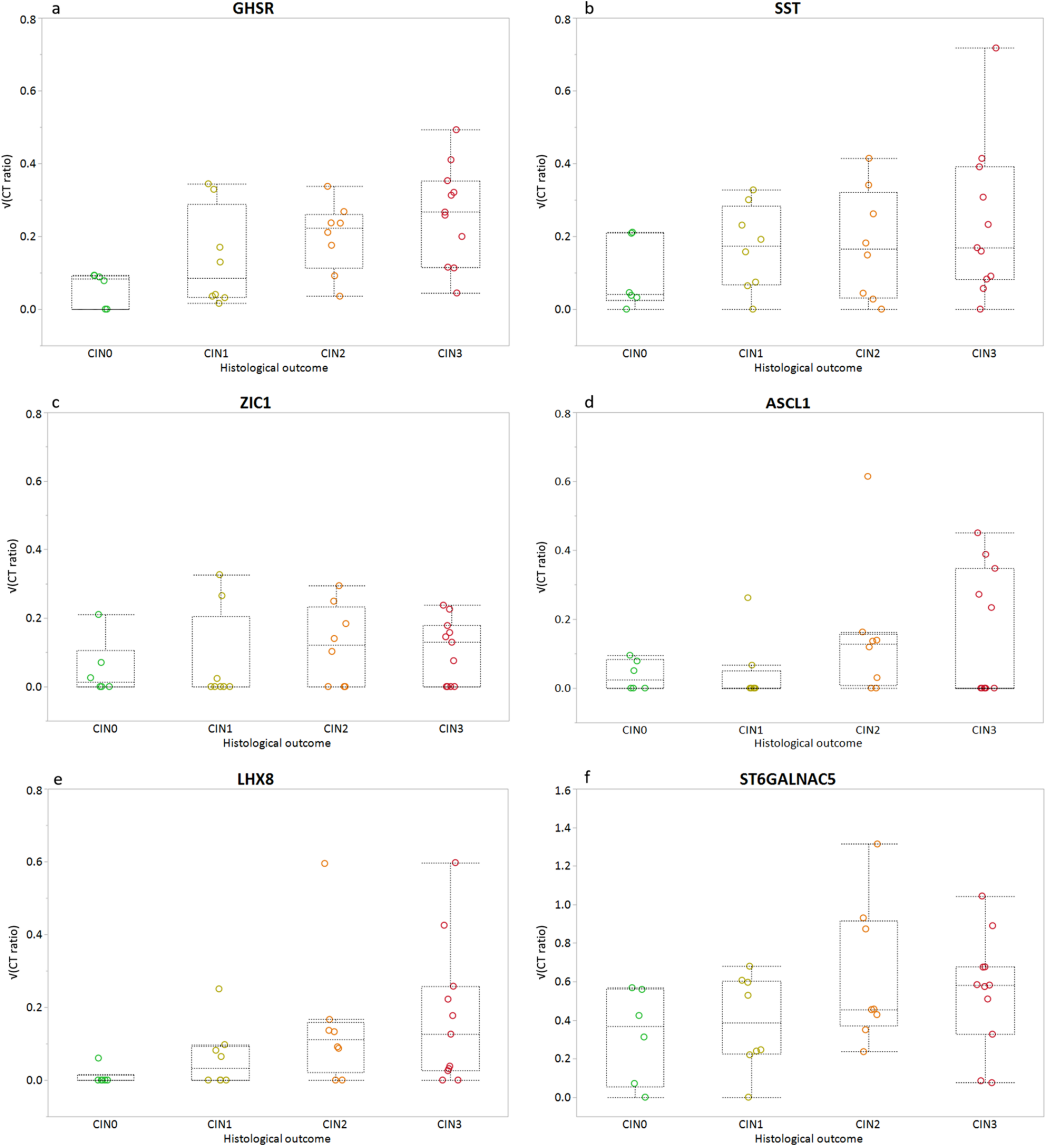
Host cell DNA methylation levels (square root transformed; y-axis) for (**a**) *GHSR*, (**b**) *SST*, (**c**) *ZIC1*, (**d**) *ASCL1*, (**e**) *LHX8*, and (**f**) *ST6GALNAC5 *according to histological outcome (x-axis); normal/CIN0 (green circles, n = 6), CIN1 (yellow circles, n = 8), CIN2 (orange circles, n = 8), and CIN3 (red circles, n = 11). Box-plots indicate median methylation levels and according interquartile ranges (25th and 75th percentile).

**Figure 4:**
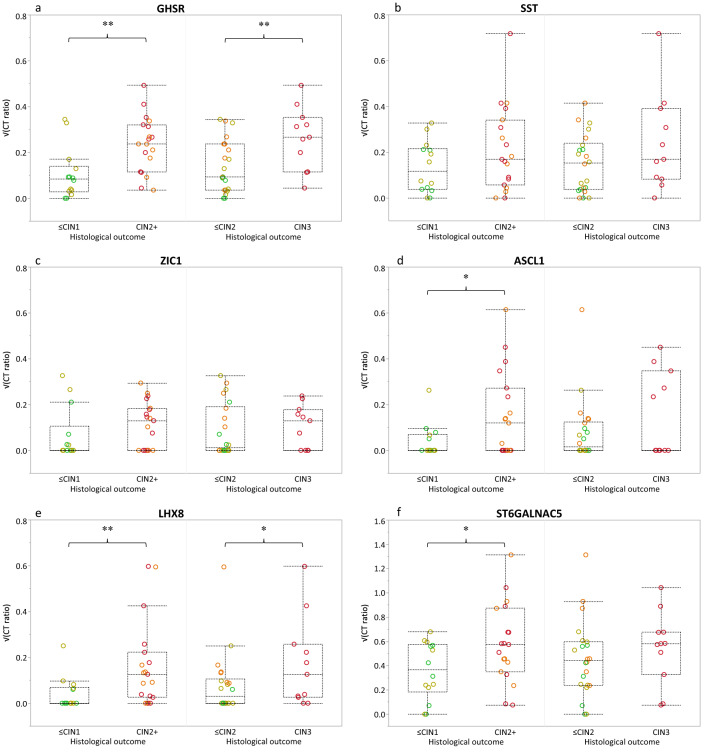
Difference in methylation ratios (square root transformed; y-axis) for normal/low-grade and high-grade cervical disease according to histological outcome (CIN2 + and CIN3; x-axis); normal/CIN0 (green circles, n = 6), CIN1 (yellow circles, n = 8), CIN2 (orange circles, n = 8), and CIN3 (red circles, n = 11). Box-plots indicate median methylation levels of (**a**) *GHSR*, (**b**) *SST*, (**c**) *ZIC1*, (**d**) *ASCL1*, (**e**) *LHX8*, and (**f**) *ST6GALNAC5* and according interquartile ranges (25th and 75th percentile). P-values (Mann Whitney U-test) indicated by a double asterisk mean that the null hypothesis (H0) is rejected (p ≤ 0.05) and that the median square root transformed methylation marker ratios between normal/low-grade and high-grade cervical disease are not equal. P-values indicated by a single asterisk indicate a trend (0.05 < p ≤ 0.10).

Secondly, we assessed the presence of the methylation marker levels in FVU of HR-HPV positive women with known histological outcome. This resulted in a set of 33 samples; 6 normal/CIN0, 8 CIN1, 8 CIN2, and 11 CIN3 (n = 33; Fig. 1). A gradual increase in methylation level was observed for *GHSR*, *SST, ASCL1,* and *LHX8*, but not for *ZIC1* and *ST6GALNAC5* (Fig. 3) with increasing severity of underlying disease. Comparison of ≤CIN1 to CIN2 + revealed increased median marker levels for all six markers in FVU, significantly elevated (p ≤ 0.05) for *GHSR* and *LHX8*. A similar trend (0.05 < p ≤ 0.10) was observed for *ASCL1* and *ST6GALNAC5* (0.05 < p ≤ 0.10) (Fig. 4 and Table 1). Comparison of ≤CIN2 to CIN3 revealed significantly elevated median methylation levels in FVU for *GHSR* (p ≤ 0.05), and a similar trend for *LHX8* (0.05 < p ≤ 0.10).

**Table 1:** Performance of individual host cell methylation markers to discern high-grade cervical disease.

Marker	P-value MWU^a^			AUC (95% CI)^b^		
	Cytology	Histology		Cytology	Histology	
	HSIL +	CIN2 +	CIN3	HSIL +	CIN2 +	CIN3
*GHSR*	0.128	0.004**	0.014**	0.622 (0.488–0.757)	0.801 (0.591–0.919)	0.769 (0.561–0.897)
*SST*	0.049**	0.251	0.221	0.658 (0.498–0.798)	0.620 (0.464–0.789)	0.634 (0.473–0.809)
*ZIC1*	0.345	0.228	0.588	0.568 (0.435–0.707)	0.620 (0.489–0.788)	0.558 (0.400–0.742)
*ASCL1*	0.125	0.068*	0.524	0.614 (0.451–0.778)	0.677 (0.497–0.819)	0.566 (0.473–0.849)
*LHX8*	0.198	0.009**	0.087*	0.598 (0.480–0.741)	0.763 (0.575–0.890)	0.682 (0.465–0.842)
*ST6GALNAC5*	0.177	0.061*	0.244	0.609 (0.465–0.725)	0.695 (0.501–0.846)	0.628 (0.453–0.804)

Performance of each marker was assessed using cytology (HSIL + , n = 89) and histology (CIN2 + and CIN3, n = 33) as reference.

^a^P-values (Mann Whitney U-test (MWU)) indicated by a double asterisk indicate that the null hypothesis (H0) is rejected (p ≤ 0.05) and that the median square root transformed methylation marker ratios between normal/low-grade and high-grade cervical disease are not equal. P-values values indicated by a single asterisk indicate a trend (0.05 < p ≤ 0.10).

^b^An estimation of the performance of each marker was evaluated by the area under the curve (AUC) and accompanying 95% confidence interval (CI).

### Triage marker performance of GHSR, SST, ZIC1, ASCL1, LHX8, and ST6GALNAC5 in first-void urine to discern high-grade disease

We have furthermore investigated the ability of these methylation markers to discriminate HR-HPV positive women with (i) cytology and (ii) biopsy confirmed high-grade disease from normal/low-grade disease. To do so, an estimation of the clinical performance of each methylation marker in FVU was evaluated by the AUC, and visualized by ROC-curves (Supplementary Fig. S1). When categorizing according to CIN2 +, AUC’s closest to 1 were observed for *GHSR* (0.80; 95% CI 0.59–0.92) and *LHX8* (0.76; 95% CI 0.58–0.89), followed by *ST6GALNAC5*, *ASCL1*, and *SST/ZIC1*, in the respective order (Supplementary Fig. S1 and Table S1). Discriminating CIN3 from ≤ CIN2 lesions yields slightly lower AUC’s, with values closest to 1 again for *GHSR* (0.77; 95% CI 0.56–0.90) and *LHX8* (0.68; 95% CI 0.47–0.84). Stratifying according to cytology (HSIL +) results in overall lower AUC’s, ranging between 0.66 (*SST*; 95% CI 0.50–0.80) and 0.57 (*ZIC1*; 95% CI 0.44–0.71), using HSIL + as endpoint (Supplementary Fig. S1 and Table S1).

## Discussion

To the best of our knowledge, this study is the first to report on the potential of methylation analysis of this panel of six host cell genes (*GHSR, SST, ZIC1, ASCL1, LHX8,* and *ST6GALNAC5*^26,29^), previously identified in HPV transformed cell lines, tissue biopsies, and cervicovaginal self-samples, in FVU. All six methylation markers showed an increase in median methylation levels in FVU for underlying disease (HSIL + and CIN2 +). Four out of six markers showed a gradual increase in median methylation levels with increasing lesion severity (normal/CIN0 through CIN3). The rise in methylation levels with increasing lesion severity was predominantly pronounced for *GHSR*, *LHX8* and *SST*, where significant increases were observed using CIN2 + /CIN3, CIN2 +, and HSIL + as endpoint, respectively.

The less pronounced difference between median methylation levels between normal/low-grade and high-grade cervical disease in FVU compared to CS/SS^26,29^ can be explained by several factors. Firstly, the investigated host cell genes, *GHSR*, *SST*, *ZIC1*, *ASCL1*, *LHX8*, and *ST6GALNAC5* were identified and validated in clinician and self-collected cervicovaginal samples. As the clinical value of methylation markers is not necessarily analogous between sample types^43^, a similar comprehensive approach including genome-wide DNA methylation profiling of FVU samples from HR-HPV positive women with normal to high-grade cervical disease and (invasive) cancer might allow us to identify methylated genes that predict high-grade cervical disease more accurately in FVU. Nevertheless, as we are using the first part of the urine void as liquid biopsy to capture cervicovaginal secretions, similarities in biomarker profile are expected. Secondly, CIN2 and CIN3 are a heterogeneous group of disease of which only a subset has a high risk of progression to cancer. Previous research has indicated that high methylation levels are associated with an advanced stage of disease and presumable high cancer progression risk. On the other hand, CIN2 and CIN3 with low methylation levels are suggested to have a low cancer progression risk^21,26^. We have no data on the duration and cancer risk of the CIN2 and CIN3 included in present study.

Others have investigated difference in host cell methylation levels in urine between cancers and controls, for which larger differentiations between groups are anticipated. One study reported increasing number of hyper methylation-positive urine samples (for ≥ 1/4 genes: *DAPK1, RARB, TWIST, CDH13*) according to lesion severity (4% CIN0/1, 28% CIN2/3/carcinoma in situ, and 62% invasive cervix carcinoma)^32^. Significant different methylation levels between cancers and controls in (first-void) urine have also been described for *ASCL1, FAM19A4, GHSR, LHX8, PHACTR3*, *PRDM14, SFRP4*, *SST*, and *ZIC1*^31,34,35^. An estimate of the diagnostic performance of the markers in our study showed similar AUC’s as those reported by van den Helder and colleagues (2020)^35^, between 0.62–0.80 and 0.56–0.77 to discriminate between CIN0/1 and CIN2 + and CIN0-2 and CIN3, respectively. Highest AUC’s were observed when comparing cancers versus controls. It is therefore of interest to investigate the discriminatory power of our methylation marker panel in FVU from cancer patients in future studies.

Furthermore, the controls used in this study to assess biomarker performance were all HR-HPV positive (w/o low-grade underlying cervical disease), which is different from other studies reporting on triage marker performance between (pre)cancers and controls with partial HR-HPV positivity^31^ or without any disease^34,35^. Smaller differences in methylation levels between cases and controls can thus be expected from our data set. However, as methylation marker(s) (panels) are proposed as a triage strategy to discern high grade disease in women with a positive HR-HPV test, we believe that the controls used in this study align well with the intended clinical use, empowering the results.

Limitations of our study need to be acknowledged, and firstly involve the relative small sample size, potentially contributing to the insignificant differences observed, and heterogeneous character of our study population. This study was designed to identify promising triage markers for HR-HPV positive women. Hereto, a referral population of women was targeted attending colposcopy, either because of first abnormal screen result, or for follow-up of a persistent infection and/or associated lesion. Histology outcomes from biopsies (only taken when indicated) and cervical conization were only available for one third of the study population. To overcome this, performance of the markers was also assessed based on cytology, notwithstanding that histology is the golden standard reference test. Including both endpoints was reinforced by good κ-agreements observed between cytology (HSIL +) and histology CIN2 + (κ: 0.60 (95% CI 0.35–0.86)) and CIN3 (κ: 0.68 (95% CI 0.42–0.94)) endpoints. The difference between methylation markers showing significance in cytology (i.e. *SST*) versus histology endpoints (i.e. *GHSR* and *LHX8*) is likely a reflection of the suboptimal performance of cytology to predict underlying disease and the relatively small sample size. For this reason and given the nature of the study being a feasibility study we did not investigate diagnostic performance of methylation marker combinations. Secondly, at the time of study initiation limited optimization experiments had been performed identifying the optimal FVU collection and pre-analytical processing method preceding methylation analysis by qMSP. Optimizations might potentially increase analytic sensitivity, and consequently distinctions between cases and controls and AUC’s. Yet, it is expected that this will not change the general conclusions from present study, showing that increases in methylation levels of host cell genes can be observed in FVU with increasing severity of underlying disease.

In summary, the data from this study propose that methylation analysis of host cell genes in FVU has the diagnostic potential to distinguish normal/low-grade from high-grade underlying cervical disease in HR-HPV infected women. Concomitantly, it is a promising liquid biopsy to offer a fully molecular screen and triage strategy based on primary HPV testing and methylation marker detection in the same sample. Its non-invasive nature, being well-accepted, and ability to be self-collected at home furthermore fortifies its use to potentially increase screening-attendance in hard-to-reach populations whilst reducing loss to follow-up. Further studies including a larger sample series with histological confirmation of underlying disease are ongoing to define the diagnostic accuracy of methylation marker testing in FVU and to extract the most discriminative methylation markers or marker combinations.

## Supplementary Information

Supplementary Information.

## Data Availability

All genotyping data generated and analysed during this study are included in this published article (and its Supplementary Information files; Supplementary Tables S1, S2 and S3). The methylation marker dataset generated and analysed during the current study is available from the corresponding author on reasonable request.
