# Supplementary material for: Triage of human papillomavirus infected women by methylation analysis in first-void urine
Source: Sci Rep. 2021 Apr 12;11:7862. doi: 10.1038/s41598-021-87329-1 (PMC8042010; doi:10.1038/s41598-021-87329-1)
Supplement: Supplementary file 1 — Supplementary Information. [file 41598_2021_87329_MOESM1_ESM.docx]

SUPPLEMENTARY MATERIAL

**Triage of human papillomavirus infected women by methylation analysis in first-void urine**

Severien Van Keer^a,*^, Annina P. van Splunter ^b^, Jade Pattyn^a^, Annemie De Smet^a^, Sereina A. Herzog^c^, Xaveer Van Ostade^d^, Wiebren A. A. Tjalma^e^, Margareta Ieven^f^, Pierre Van Damme^a^, Renske D. M. Steenbergen^b^, Alex Vorsters^a^

*^a^Centre for the Evaluation of Vaccination (CEV); Vaccine & Infectious Disease Institute (VAXINFECTIO); Faculty of Medicine and Health Sciences; University of Antwerp; Universiteitsplein 1, 2610 Wilrijk (Belgium)*

*^b^Amsterdam UMC; Vrije Universiteit Amsterdam; Pathology, Cancer Center Amsterdam; De Boelelaan 1117, 1081 HV Amsterdam (The Netherlands)*

*^c^Centre for Health Economics Research and Modelling Infectious Diseases (CHERMID); Vaccine & Infectious Disease Institute (VAXINFECTIO); Faculty of Medicine and Health Sciences; University of Antwerp; Universiteitsplein 1, 2610 Wilrijk (Belgium)*

*^d^Laboratory of Proteinscience, Proteomics & Epigenetic Signalling (PPES); Faculty of Pharmaceutical, Biomedical and Veterinary Sciences; University of Antwerp; Universiteitsplein 1, 2610 Wilrijk (Belgium)*

*^e^Multidisciplinary Breast Clinic, Unit Gynaecologic Oncology; Department of Obstetrics and Gynaecology; Antwerp University Hospital (UZA); Wilrijkstraat 10, 2650 Edegem (Belgium); Molecular Imaging, Pathology, Radiotherapy, Oncology (MIPRO); Faculty of Medicine and Health Sciences; University of Antwerp; Universiteitsplein 1, 2610 Wilrijk (Belgium)*

*^f^Laboratory of Medical Microbiology (LMM); Vaccine & Infectious Disease Institute (VAXINFECTIO); Faculty of Medicine and Health Sciences; University of Antwerp; Universiteitsplein 1, 2610 Wilrijk (Belgium)*

**Corresponding author: Severien Van Keer; Campus Drie Eiken, Building S2, Universiteitsplein 1, 2610 Wilrijk (Belgium); Tel. +32(0)3/265.90.10;* [*severien.vankeer@uantwerpen.be*](mailto:severien.vankeer@uantwerpen.be)

**Supplementary Table 1.** Study population characteristics.

| **Characteristic** | **Total**  (n=119) | | **(p)HR-HPV positive w/cytology endpoint** (n=89) | | **(p)HR-HPV positive w/histology endpoint** (n=33) | |
| --- | --- | --- | --- | --- | --- | --- |
|  |  |  |  |  |  |  |
| **Demographics** |  |  |  |  |  |  |
| Study participants (n, %) | 119 | 100 | 89 | 100 | 33 | 100 |
| Age, years (median, IQR) | 36 | 29-44 | 36 | 29-44 | 35 | 29-44 |
| Smoking (n, valid %)^a,b^ | 36 | 31 | 27 | 31 | 12 | 36 |
| Age sexual debut, years (median, IQR)^a,b,c^ | 17 | 16-18 | 17 | 16-18 | 17 | 16-18 |
| Parous (n, valid %)^a,b,c^ | 53 | 48 | 38 | 47 | 17 | 53 |
| Primiparous (n, % according to parous women) | 21 | 40 | 17 | 45 | 7 | 41 |
| Multiparous^d^ (n, % according to parous women) | 32 | 60 | 21 | 55 | 10 | 59 |
| Oral contraceptive use (n, valid %)^a,b^ | 38 | 32 | 27 | 31 | 10 | 30 |
| Age first pap smear, years (median, IQR)^a,b,c^ | 19 | 18-19 | 19 | 18-25 | 20 | 18-26 |
|  |  |  |  |  |  |  |
| **Disease outcomes** | **n** | **%** | **n** | **%** | **n** | **%** |
| ***Cytology results*** *(n, %)^a,c^* | *115* | *97* | *89* | *100* | *31* | *94* |
| NILM (n, valid %) | 59 | 51 | 40 | 45 | 9 | 30 |
| AGC (n, valid %) | 1 | 1 | 0 | 0 | 0 | 0 |
| ASC-US (n, valid %) | 20 | 17 | 19 | 21 | 5 | 16 |
| LSIL (n, valid %) | 19 | 17 | 14 | 16 | 5 | 16 |
| ASC-H (n, valid %) | 8 | 7 | 8 | 9 | 6 | 19 |
| HSIL (n, valid %) | 8 | 7 | 8 | 9 | 6 | 19 |
|  |  |  |  |  |  |  |
| ***Colposcopy results*** *(n, %)^a,b,c^* | *114* | *96* | *85* | *96* | *29* | *88* |
| Normal (n, valid %) | 35 | 31 | 24 | 28 | 4 | 14 |
| Low-grade (n, valid %) | 60 | 52 | 44 | 52 | 10 | 34 |
| High-grade (n, valid %) | 19 | 17 | 17 | 20 | 15 | 52 |
|  |  |  |  |  |  |  |
| ***Histology results*** *(n, %)^a,b^* | *38* | *32* | *31* | *35* | *33* | *100* |
| CIN0 (n, valid %) | 7 | 18 | 6 | 19 | 6 | 18 |
| CIN1 (n, valid %) | 12 | 32 | 8 | 26 | 8 | 24 |
| CIN2 (n, valid %) | 8 | 21 | 6 | 19 | 8 | 24 |
| CIN3 (n, valid %) | 11 | 29 | 11 | 36 | 11 | 33 |
|  |  |  |  |  |  |  |
| ***HPV DNA results in clinician-collected cervical samples*** *(n, %)^a,b,c^* | *114* | *96* | *88* | *99* | *30* | *91* |
| (p)HR-HPV positive (n, valid %) | 78 | 68 | 78 | 89 | 28 | 93 |
| Multiple (p)HR-HPV infections (n, % according to (p)HR-HPV positives) | 30 | 38 | 30 | 38 | 10 | 36 |
| HPV16/18 positive (n, % according to (p)HR-HPV positives) | 27 | 35 | 27 | 35 | 14 | 50 |
| HPV16 positive (n, % according to (p)HR-HPV positives) | 22 | 28 | 22 | 28 | 12 | 43 |
| HPV18 positive (n, % according to (p)HR-HPV positives) | 6 | 8 | 6 | 8 | 2 | 7 |
|  |  |  |  |  |  |  |
| ***HPV DNA results in first-void urine*** *(n, %)* | *119* | *100* | *89* | *100* | *33* | *100* |
| (p)HR-HPV positive (n, valid %) | 86 | 72 | 82 | 92 | 32 | 97 |
| Multiple (p)HR-HPV infections (n, % according to (p)HR-HPV positives) | 47 | 55 | 45 | 55 | 15 | 47 |
| HPV16/18 positive (n, % according to (p)HR-HPV positives) | 31 | 36 | 29 | 35 | 17 | 53 |
| HPV16 positive (n, % according to (p)HR-HPV positives) | 26 | 30 | 24 | 29 | 15 | 47 |
| HPV18 positive (n, % according to (p)HR-HPV positives) | 7 | 8 | 7 | 9 | 2 | 6 |

n: number; IQR: interquartile range; SE: standard error; NILM: negative for intraepithelial lesion and malignancy; AGC: atypical glandular cells of undetermined significance; ASC-US: atypical squamous cells of undetermined significance; ASC-H: atypical squamous cells, cannot exclude HSIL; LSIL: low-grade squamous intraepithelial lesion; HSIL: high-grade squamous intraepithelial lesion; CIN_x_: cervical intraepithelial neoplasia grade x; Probable (p) high-risk (HR) (HPV68) and HR-HPV genotypes (HPV16/18/31/33/35/39/45/51/52/56/58/59)^1^. Total number n available per variable for; ^a^total study population: smoking (117), age sexual debut (115), parity (110), oral contraceptive use (117), age first pap smear (113), cytology (115), colposcopy (114), histology (38), HPV DNA in clinician-collected cervical samples (114); ^b^(p)HR-HPV positives with (w/)cytology: smoking (88), age sexual debut (86), parity (81), oral contraceptive use (87), age first pap smear (83), colposcopy (85), histology (31), HPV DNA in clinician-collected cervical samples (88); and ^c^(p)HR-HPV positives w/histology: age sexual debut (31), parity (32), age first pap smear (29), cytology (31), colposcopy (29), HPV DNA in clinician-collected cervical samples (30). ^d^Multiparous: minimum 2; maximum 6 (for total study population and subgroups)

**Supplementary Table 2.** Raw human (h) and HPV DNA genotyping outcomes (copies/µl DNA extract) in first-void urine using the Riatol quantitative PCR HPV genotyping assay.

| **ID** | **hDNA**  **(ng/µl)** | **First-void urine (copies/µl)** | | | | | | | | | | | | | | | | | |
| --- | --- | --- | --- | --- | --- | --- | --- | --- | --- | --- | --- | --- | --- | --- | --- | --- | --- | --- | --- |
|  |  | **HPV6** | **HPV11** | **HPV16** | **HPV18** | **HPV31** | **HPV33** | **HPV35** | **HPV39** | **HPV45** | **HPV51** | **HPV52** | **HPV53** | **HPV56** | **HPV58** | **HPV59** | **HPV66** | **HPV67** | **HPV68** |
| **1** | 4.61 | 0.00 | 0.00 | 5.54E+03 | 0.00 | 0.00 | 0.00 | 0.00 | 0.00 | 0.00 | 0.00 | 0.00 | 0.00 | 0.00 | 0.00 | 0.00 | 0.00 | 0.00 | 4.93E+03 |
| **2** | 8.23 | 0.00 | 0.00 | 0.00 | 0.00 | 0.00 | 0.00 | 0.00 | 0.00 | 0.00 | 0.00 | 0.00 | 0.00 | 0.00 | 0.00 | 0.00 | 0.00 | 1.99E+07 | 0.00 |
| **3** | 7.92 | 0.00 | 0.00 | 0.00 | 0.00 | 2.28E+07 | 0.00 | 0.00 | 0.00 | 0.00 | 0.00 | 4.33E+03 | 0.00 | 0.00 | 0.00 | 0.00 | 0.00 | 0.00 | 0.00 |
| **4** | 1.20E+02 | 2.68E+06 | 0.00 | 0.00 | 0.00 | 0.00 | 0.00 | 0.00 | 0.00 | 0.00 | 1.64E+07 | 9.09E+08 | 0.00 | 0.00 | 0.00 | 0.00 | 0.00 | 0.00 | 0.00 |
| **5** | 14.15 | 0.00 | 0.00 | 1.58E+06 | 7.71E+05 | 0.00 | 0.00 | 0.00 | 0.00 | 0.00 | 0.00 | 0.00 | 0.00 | 0.00 | 0.00 | 0.00 | 5.86E+06 | 0.00 | 1.97E+07 |
| **6** | 4.36 | 0.00 | 0.00 | 0.00 | 0.00 | 0.00 | 0.00 | 0.00 | 7.62E+04 | 0.00 | 0.00 | 0.00 | 0.00 | 0.00 | 0.00 | 0.00 | 0.00 | 0.00 | 1.01E+06 |
| **7** | 61.94 | 0.00 | 0.00 | 0.00 | 0.00 | 2.44E+05 | 0.00 | 0.00 | 0.00 | 0.00 | 7.18E+06 | 0.00 | 1.73E+06 | 0.00 | 0.00 | 0.00 | 0.00 | 0.00 | 0.00 |
| **8** | 10.76 | 0.00 | 0.00 | 0.00 | 0.00 | 0.00 | 0.00 | 0.00 | 0.00 | 0.00 | 0.00 | 0.00 | 0.00 | 0.00 | 0.00 | 0.00 | 0.00 | 0.00 | 1.03E+05 |
| **9** | 57.08 | 0.00 | 0.00 | 0.00 | 0.00 | 0.00 | 0.00 | 0.00 | 0.00 | 0.00 | 0.00 | 0.00 | 0.00 | 0.00 | 0.00 | 0.00 | 0.00 | 0.00 | 0.00 |
| **10** | 24.37 | 0.00 | 0.00 | 0.00 | 0.00 | 0.00 | 0.00 | 0.00 | 0.00 | 0.00 | 0.00 | 0.00 | 0.00 | 0.00 | 0.00 | 0.00 | 0.00 | 0.00 | 0.00 |
| **11** | 7.77 | 0.00 | 0.00 | 0.00 | 0.00 | 0.00 | 0.00 | 0.00 | 0.00 | 0.00 | 0.00 | 0.00 | 0.00 | 0.00 | 0.00 | 0.00 | 1.38E+07 | 0.00 | 0.00 |
| **12** | 20.95 | 0.00 | 0.00 | 0.00 | 0.00 | 6.73E+07 | 9.48E+06 | 0.00 | 1.46E+04 | 0.00 | 0.00 | 0.00 | 0.00 | 0.00 | 1.59E+08 | 4.59E+06 | 0.00 | 0.00 | 0.00 |
| **13** | 26.64 | 0.00 | 0.00 | 0.00 | 0.00 | 0.00 | 0.00 | 0.00 | 0.00 | 0.00 | 0.00 | 0.00 | 0.00 | 0.00 | 0.00 | 0.00 | 0.00 | 0.00 | 0.00 |
| **14** | 13.04 | 0.00 | 0.00 | 0.00 | 0.00 | 0.00 | 0.00 | 0.00 | 0.00 | 0.00 | 0.00 | 0.00 | 0.00 | 0.00 | 0.00 | 0.00 | 0.00 | 0.00 | 0.00 |
| **15** | 33.35 | 0.00 | 0.00 | 0.00 | 0.00 | 2.02E+08 | 0.00 | 0.00 | 0.00 | 6.57E+04 | 0.00 | 0.00 | 0.00 | 0.00 | 0.00 | 0.00 | 0.00 | 0.00 | 0.00 |
| **16** | 30.63 | 0.00 | 0.00 | 8.25E+04 | 0.00 | 0.00 | 0.00 | 6.75E+05 | 0.00 | 0.00 | 0.00 | 6.68E+05 | 0.00 | 0.00 | 0.00 | 0.00 | 0.00 | 0.00 | 0.00 |
| **17** | 4.60 | 0.00 | 0.00 | 0.00 | 0.00 | 0.00 | 0.00 | 0.00 | 0.00 | 0.00 | 0.00 | 0.00 | 0.00 | 0.00 | 0.00 | 0.00 | 0.00 | 0.00 | 0.00 |
| **18** | 75.08 | 0.00 | 0.00 | 0.00 | 0.00 | 0.00 | 1.77E+04 | 0.00 | 4.82E+05 | 0.00 | 0.00 | 0.00 | 0.00 | 0.00 | 0.00 | 1.08E+06 | 7.05E+05 | 0.00 | 0.00 |
| **19** | 19.39 | 0.00 | 0.00 | 0.00 | 0.00 | 0.00 | 0.00 | 0.00 | 0.00 | 0.00 | 0.00 | 0.00 | 0.00 | 0.00 | 0.00 | 0.00 | 0.00 | 0.00 | 0.00 |
| **20** | 5.68 | 0.00 | 0.00 | 0.00 | 0.00 | 0.00 | 0.00 | 0.00 | 0.00 | 0.00 | 0.00 | 0.00 | 0.00 | 0.00 | 0.00 | 0.00 | 0.00 | 0.00 | 0.00 |
| **21** | 28.95 | 0.00 | 0.00 | 0.00 | 0.00 | 2.19E+08 | 0.00 | 0.00 | 0.00 | 0.00 | 0.00 | 0.00 | 0.00 | 0.00 | 0.00 | 0.00 | 0.00 | 0.00 | 5.40E+04 |
| **22** | 8.29 | 0.00 | 0.00 | 0.00 | 0.00 | 0.00 | 8.31E+04 | 0.00 | 0.00 | 0.00 | 0.00 | 0.00 | 0.00 | 0.00 | 0.00 | 0.00 | 0.00 | 0.00 | 2.50E+05 |
| **23** | 7.62 | 3.97E+05 | 1.16E+05 | 5.79E+06 | 0.00 | 0.00 | 0.00 | 1.40E+04 | 0.00 | 0.00 | 0.00 | 0.00 | 1.00E+06 | 0.00 | 0.00 | 0.00 | 0.00 | 3.74E+03 | 1.83E+07 |
| **24** | 21.08 | 0.00 | 0.00 | 0.00 | 0.00 | 0.00 | 0.00 | 0.00 | 0.00 | 0.00 | 0.00 | 0.00 | 0.00 | 0.00 | 0.00 | 0.00 | 0.00 | 0.00 | 0.00 |
| **25** | 31.63 | 0.00 | 0.00 | 0.00 | 0.00 | 0.00 | 0.00 | 0.00 | 0.00 | 0.00 | 0.00 | 0.00 | 0.00 | 0.00 | 0.00 | 0.00 | 0.00 | 0.00 | 0.00 |
| **26** | 29.07 | 0.00 | 0.00 | 0.00 | 0.00 | 0.00 | 0.00 | 0.00 | 1.69E+05 | 3.72E+05 | 7.86E+07 | 0.00 | 0.00 | 0.00 | 0.00 | 6.55E+07 | 2.20E+08 | 0.00 | 0.00 |
| **27** | 64.41 | 0.00 | 0.00 | 0.00 | 0.00 | 0.00 | 0.00 | 0.00 | 0.00 | 0.00 | 0.00 | 0.00 | 0.00 | 0.00 | 0.00 | 0.00 | 0.00 | 0.00 | 0.00 |
| **28** | 2.76 | 0.00 | 0.00 | 0.00 | 0.00 | 0.00 | 0.00 | 0.00 | 0.00 | 0.00 | 0.00 | 0.00 | 0.00 | 0.00 | 0.00 | 0.00 | 0.00 | 0.00 | 0.00 |
| **29** | 32.89 | 0.00 | 0.00 | 0.00 | 0.00 | 0.00 | 0.00 | 0.00 | 0.00 | 0.00 | 0.00 | 0.00 | 0.00 | 0.00 | 0.00 | 0.00 | 0.00 | 0.00 | 0.00 |
| **30** | 6.69 | 0.00 | 0.00 | 0.00 | 0.00 | 0.00 | 0.00 | 0.00 | 0.00 | 0.00 | 0.00 | 0.00 | 0.00 | 0.00 | 2.52E+05 | 0.00 | 0.00 | 0.00 | 0.00 |
| **31** | 35.99 | 0.00 | 0.00 | 0.00 | 0.00 | 0.00 | 4.25E+04 | 0.00 | 0.00 | 0.00 | 0.00 | 0.00 | 0.00 | 0.00 | 0.00 | 0.00 | 0.00 | 0.00 | 0.00 |
| **32** | 28.36 | 0.00 | 0.00 | 0.00 | 0.00 | 2.55E+07 | 0.00 | 0.00 | 0.00 | 0.00 | 4.78E+04 | 0.00 | 0.00 | 0.00 | 0.00 | 0.00 | 0.00 | 6.59E+02 | 0.00 |
| **33** | 18.52 | 0.00 | 0.00 | 0.00 | 0.00 | 1.75E+04 | 0.00 | 0.00 | 0.00 | 0.00 | 0.00 | 0.00 | 0.00 | 0.00 | 2.99E+03 | 0.00 | 0.00 | 0.00 | 0.00 |
| **34** | 5.41 | 0.00 | 0.00 | 0.00 | 0.00 | 0.00 | 0.00 | 0.00 | 0.00 | 0.00 | 0.00 | 0.00 | 0.00 | 0.00 | 0.00 | 0.00 | 0.00 | 0.00 | 0.00 |
| **35** | 27.75 | 0.00 | 0.00 | 0.00 | 0.00 | 0.00 | 0.00 | 0.00 | 0.00 | 0.00 | 0.00 | 0.00 | 29.01 | 0.00 | 0.00 | 4.29E+05 | 0.00 | 0.00 | 0.00 |
| **36** | 30.89 | 0.00 | 0.00 | 0.00 | 0.00 | 3.82E+07 | 0.00 | 0.00 | 0.00 | 0.00 | 0.00 | 0.00 | 0.00 | 0.00 | 0.00 | 0.00 | 0.00 | 0.00 | 0.00 |
| **37** | 40.87 | 0.00 | 0.00 | 6.91E+05 | 0.00 | 0.00 | 0.00 | 0.00 | 0.00 | 0.00 | 0.00 | 0.00 | 0.00 | 0.00 | 0.00 | 0.00 | 0.00 | 0.00 | 0.00 |
| **38** | 1.98 | 0.00 | 0.00 | 4.70E+03 | 0.00 | 0.00 | 0.00 | 0.00 | 0.00 | 0.00 | 1.11E+04 | 0.00 | 0.00 | 0.00 | 0.00 | 0.00 | 0.00 | 0.00 | 0.00 |
| **39** | 27.72 | 0.00 | 0.00 | 0.00 | 0.00 | 0.00 | 0.00 | 0.00 | 0.00 | 0.00 | 3.82E+06 | 0.00 | 0.00 | 0.00 | 0.00 | 0.00 | 4.30E+07 | 0.00 | 0.00 |
| **40** | 8.71 | 0.00 | 0.00 | 0.00 | 0.00 | 1.65E+05 | 0.00 | 0.00 | 0.00 | 0.00 | 0.00 | 0.00 | 0.00 | 0.00 | 0.00 | 0.00 | 0.00 | 0.00 | 0.00 |
| **41** | 19.37 | 0.00 | 0.00 | 0.00 | 0.00 | 0.00 | 0.00 | 0.00 | 4.69E+06 | 0.00 | 3.62E+05 | 1.26E+04 | 0.00 | 0.00 | 1.75E+07 | 0.00 | 0.00 | 0.00 | 0.00 |
| **42** | 3.23 | 0.00 | 0.00 | 0.00 | 0.00 | 0.00 | 0.00 | 0.00 | 0.00 | 0.00 | 0.00 | 0.00 | 0.00 | 0.00 | 0.00 | 0.00 | 0.00 | 0.00 | 0.00 |
| **43** | 12.06 | 0.00 | 0.00 | 0.00 | 0.00 | 0.00 | 0.00 | 0.00 | 0.00 | 0.00 | 0.00 | 0.00 | 23.22 | 4.08E+05 | 0.00 | 0.00 | 0.00 | 0.00 | 0.00 |
| **44** | 1.06E+02 | 0.00 | 0.00 | 0.00 | 0.00 | 0.00 | 0.00 | 0.00 | 0.00 | 0.00 | 1.31E+06 | 0.00 | 0.00 | 0.00 | 0.00 | 0.00 | 0.00 | 0.00 | 0.00 |
| **45** | 11.78 | 0.00 | 0.00 | 1.41E+06 | 0.00 | 0.00 | 0.00 | 0.00 | 0.00 | 0.00 | 0.00 | 0.00 | 0.00 | 0.00 | 0.00 | 0.00 | 0.00 | 9.64E+03 | 0.00 |
| **46** | 3.79 | 0.00 | 1.27E+04 | 0.00 | 0.00 | 0.00 | 0.00 | 0.00 | 0.00 | 2.69E+06 | 0.00 | 0.00 | 0.00 | 0.00 | 0.00 | 4.33E+03 | 0.00 | 0.00 | 8.90E+04 |
| **47** | 61.16 | 0.00 | 0.00 | 0.00 | 0.00 | 0.00 | 0.00 | 0.00 | 0.00 | 0.00 | 0.00 | 0.00 | 0.00 | 0.00 | 0.00 | 0.00 | 0.00 | 0.00 | 0.00 |
| **48** | 6.19 | 0.00 | 0.00 | 735.53 | 0.00 | 0.00 | 0.00 | 0.00 | 0.00 | 0.00 | 0.00 | 0.00 | 0.00 | 0.00 | 0.00 | 0.00 | 0.00 | 0.00 | 0.00 |
| **49** | 29.62 | 0.00 | 0.00 | 0.00 | 0.00 | 2.23E+05 | 0.00 | 5.79E+06 | 0.00 | 0.00 | 0.00 | 0.00 | 0.00 | 1.13E+05 | 0.00 | 0.00 | 0.00 | 0.00 | 0.00 |
| **50** | 3.71 | 0.00 | 0.00 | 0.00 | 0.00 | 0.00 | 0.00 | 0.00 | 0.00 | 0.00 | 0.00 | 0.00 | 0.00 | 0.00 | 0.00 | 0.00 | 2.64E+03 | 0.00 | 0.00 |
| **51** | 1.08E+02 | 0.00 | 0.00 | 0.00 | 0.00 | 0.00 | 0.00 | 0.00 | 0.00 | 2.46E+05 | 0.00 | 0.00 | 1.64E+08 | 0.00 | 0.00 | 0.00 | 0.00 | 9.39E+06 | 0.00 |
| **52** | 5.53 | 0.00 | 0.00 | 0.00 | 0.00 | 0.00 | 0.00 | 0.00 | 0.00 | 0.00 | 0.00 | 0.00 | 0.00 | 1.40E+06 | 0.00 | 0.00 | 0.00 | 0.00 | 0.00 |
| **53** | 92.29 | 0.00 | 0.00 | 9.71E+06 | 0.00 | 0.00 | 0.00 | 0.00 | 0.00 | 0.00 | 0.00 | 0.00 | 0.00 | 0.00 | 0.00 | 0.00 | 0.00 | 0.00 | 0.00 |
| **54** | 2.17 | 0.00 | 0.00 | 0.00 | 0.00 | 0.00 | 0.00 | 0.00 | 1.20E+06 | 0.00 | 0.00 | 0.00 | 6.39E+06 | 0.00 | 0.00 | 0.00 | 0.00 | 88.00 | 0.00 |
| **55** | 45.04 | 0.00 | 0.00 | 8.26E+06 | 0.00 | 0.00 | 0.00 | 0.00 | 0.00 | 0.00 | 0.00 | 0.00 | 0.00 | 0.00 | 0.00 | 0.00 | 0.00 | 0.00 | 0.00 |
| **56** | 42.30 | 0.00 | 0.00 | 0.00 | 0.00 | 0.00 | 0.00 | 0.00 | 0.00 | 0.00 | 0.00 | 0.00 | 0.00 | 0.00 | 0.00 | 0.00 | 0.00 | 0.00 | 0.00 |
| **57** | 85.95 | 0.00 | 0.00 | 0.00 | 0.00 | 5.73E+08 | 0.00 | 0.00 | 0.00 | 0.00 | 0.00 | 0.00 | 0.00 | 0.00 | 0.00 | 0.00 | 0.00 | 0.00 | 0.00 |
| **58** | 3.44 | 0.00 | 0.00 | 1.37E+04 | 0.00 | 0.00 | 0.00 | 0.00 | 0.00 | 0.00 | 6.16E+03 | 0.00 | 0.00 | 4.69E+03 | 0.00 | 0.00 | 0.00 | 0.00 | 0.00 |
| **59** | 17.08 | 0.00 | 0.00 | 0.00 | 0.00 | 0.00 | 0.00 | 0.00 | 0.00 | 0.00 | 0.00 | 0.00 | 6.21E+05 | 0.00 | 0.00 | 0.00 | 0.00 | 0.00 | 0.00 |
| **60** | 30.39 | 0.00 | 0.00 | 2.30E+08 | 0.00 | 0.00 | 0.00 | 0.00 | 0.00 | 0.00 | 0.00 | 0.00 | 0.00 | 0.00 | 0.00 | 0.00 | 0.00 | 0.00 | 0.00 |
| **61** | 51.27 | 0.00 | 0.00 | 7.28E+05 | 0.00 | 0.00 | 0.00 | 0.00 | 0.00 | 0.00 | 0.00 | 2.16E+05 | 1.17 | 0.00 | 0.00 | 0.00 | 0.00 | 0.00 | 0.00 |
| **62** | 41.06 | 0.00 | 0.00 | 0.00 | 0.00 | 1.54E+08 | 0.00 | 0.00 | 0.00 | 0.00 | 0.00 | 0.00 | 0.00 | 0.00 | 0.00 | 0.00 | 0.00 | 0.00 | 3.39E+05 |
| **63** | 9.57 | 0.00 | 0.00 | 0.00 | 8.96E+04 | 0.00 | 0.00 | 0.00 | 0.00 | 0.00 | 0.00 | 0.00 | 0.00 | 0.00 | 0.00 | 0.00 | 0.00 | 0.00 | 0.00 |
| **64** | 19.38 | 0.00 | 0.00 | 0.00 | 0.00 | 2.69E+06 | 0.00 | 0.00 | 1.02E+05 | 0.00 | 0.00 | 0.00 | 0.00 | 0.00 | 0.00 | 0.00 | 0.00 | 0.00 | 0.00 |
| **65** | 1.02 | 0.00 | 0.00 | 0.00 | 1.46E+06 | 0.00 | 0.00 | 0.00 | 0.00 | 0.00 | 0.00 | 0.00 | 0.00 | 0.00 | 0.00 | 0.00 | 0.00 | 0.00 | 0.00 |
| **66** | 1.13E+02 | 0.00 | 0.00 | 0.00 | 6.62E+05 | 0.00 | 0.00 | 0.00 | 0.00 | 0.00 | 0.00 | 0.00 | 0.00 | 0.00 | 0.00 | 0.00 | 0.00 | 0.00 | 0.00 |
| **67** | 11.46 | 0.00 | 0.00 | 0.00 | 0.00 | 5.64E+04 | 0.00 | 0.00 | 0.00 | 0.00 | 0.00 | 0.00 | 0.00 | 0.00 | 0.00 | 0.00 | 2.43E+05 | 0.00 | 0.00 |
| **68** | 6.98 | 0.00 | 0.00 | 0.00 | 0.00 | 0.00 | 0.00 | 0.00 | 0.00 | 0.00 | 0.00 | 0.00 | 0.00 | 0.00 | 0.00 | 0.00 | 5.29E+07 | 0.00 | 0.00 |
| **69** | 4.17 | 0.00 | 0.00 | 8.53E+03 | 0.00 | 0.00 | 0.00 | 0.00 | 0.00 | 1.89E+04 | 0.00 | 0.00 | 0.00 | 0.00 | 0.00 | 0.00 | 0.00 | 0.00 | 0.00 |
| **70** | 19.86 | 0.00 | 0.00 | 0.00 | 0.00 | 5.60E+04 | 0.00 | 0.00 | 0.00 | 0.00 | 0.00 | 0.00 | 0.00 | 0.00 | 0.00 | 0.00 | 0.00 | 0.00 | 0.00 |
| **71** | 14.05 | 0.00 | 0.00 | 1.85E+07 | 3.54E+04 | 0.00 | 0.00 | 0.00 | 0.00 | 0.00 | 0.00 | 0.00 | 0.00 | 0.00 | 4.18E+05 | 0.00 | 0.00 | 0.00 | 0.00 |
| **72** | 3.94 | 0.00 | 0.00 | 0.00 | 0.00 | 0.00 | 0.00 | 0.00 | 0.00 | 0.00 | 3.37E+05 | 0.00 | 0.00 | 0.00 | 0.00 | 0.00 | 0.00 | 0.00 | 4.01E+05 |
| **73** | 11.17 | 0.00 | 0.00 | 0.00 | 0.00 | 3.87E+05 | 0.00 | 0.00 | 0.00 | 0.00 | 0.00 | 0.00 | 1.79E+05 | 0.00 | 0.00 | 0.00 | 0.00 | 0.00 | 2.00E+05 |
| **74** | 2.91 | 0.00 | 0.00 | 0.00 | 0.00 | 0.00 | 0.00 | 0.00 | 0.00 | 0.00 | 7.65E+05 | 0.00 | 0.00 | 0.00 | 0.00 | 0.00 | 0.00 | 0.00 | 0.00 |
| **75** | 1.97 | 0.00 | 0.00 | 0.00 | 0.00 | 0.00 | 0.00 | 0.00 | 0.00 | 0.00 | 0.00 | 0.00 | 0.00 | 0.00 | 0.00 | 0.00 | 0.00 | 0.00 | 0.00 |
| **76** | 53.11 | 0.00 | 0.00 | 0.00 | 0.00 | 0.00 | 0.00 | 0.00 | 0.00 | 0.00 | 0.00 | 0.00 | 0.00 | 0.00 | 0.00 | 0.00 | 0.00 | 0.00 | 9.83E+04 |
| **77** | 23.15 | 0.00 | 0.00 | 5.25E+04 | 0.00 | 0.00 | 0.00 | 0.00 | 0.00 | 0.00 | 0.00 | 0.00 | 0.00 | 0.00 | 0.00 | 0.00 | 0.00 | 5.15E+03 | 0.00 |
| **78** | 21.83 | 0.00 | 0.00 | 0.00 | 0.00 | 1.65E+08 | 0.00 | 0.00 | 0.00 | 0.00 | 0.00 | 0.00 | 0.00 | 0.00 | 0.00 | 0.00 | 0.00 | 0.00 | 0.00 |
| **79** | 7.87 | 0.00 | 0.00 | 0.00 | 0.00 | 0.00 | 0.00 | 0.00 | 0.00 | 3.79E+04 | 0.00 | 0.00 | 0.00 | 0.00 | 0.00 | 3.82E+05 | 4.78E+07 | 0.00 | 0.00 |
| **80** | 5.75 | 0.00 | 0.00 | 0.00 | 0.00 | 0.00 | 0.00 | 0.00 | 0.00 | 0.00 | 0.00 | 0.00 | 0.00 | 0.00 | 0.00 | 0.00 | 0.00 | 2.45E+03 | 0.00 |
| **81** | 32.59 | 0.00 | 0.00 | 3.25E+07 | 0.00 | 0.00 | 0.00 | 0.00 | 0.00 | 0.00 | 3.17E+05 | 0.00 | 0.00 | 9.81E+04 | 0.00 | 0.00 | 5.10E+07 | 0.00 | 0.00 |
| **82** | 12.00 | 0.00 | 0.00 | 4.32E+03 | 0.00 | 0.00 | 0.00 | 0.00 | 0.00 | 0.00 | 0.00 | 0.00 | 0.00 | 0.00 | 0.00 | 0.00 | 0.00 | 0.00 | 0.00 |
| **83** | 43.80 | 0.00 | 0.00 | 1.33E+05 | 0.00 | 0.00 | 0.00 | 0.00 | 0.00 | 0.00 | 0.00 | 0.00 | 0.00 | 0.00 | 0.00 | 0.00 | 0.00 | 0.00 | 2.96E+05 |
| **84** | 4.27 | 0.00 | 0.00 | 0.00 | 0.00 | 0.00 | 0.00 | 0.00 | 0.00 | 0.00 | 0.00 | 0.00 | 3.23E+07 | 0.00 | 0.00 | 0.00 | 0.00 | 0.00 | 0.00 |
| **85** | 8.34 | 0.00 | 0.00 | 0.00 | 0.00 | 1.56E+05 | 0.00 | 0.00 | 0.00 | 0.00 | 0.00 | 0.00 | 0.00 | 0.00 | 0.00 | 0.00 | 0.00 | 0.00 | 0.00 |
| **86** | 21.41 | 0.00 | 0.00 | 0.00 | 0.00 | 1.62E+08 | 0.00 | 1.56E+06 | 0.00 | 0.00 | 0.00 | 0.00 | 5.27E+04 | 0.00 | 0.00 | 0.00 | 0.00 | 0.00 | 0.00 |
| **87** | 40.39 | 0.00 | 0.00 | 0.00 | 0.00 | 0.00 | 0.00 | 0.00 | 0.00 | 0.00 | 0.00 | 2.54E+05 | 0.00 | 0.00 | 0.00 | 4.46E+04 | 0.00 | 0.00 | 1.10E+06 |
| **88** | 2.34 | 0.00 | 0.00 | 0.00 | 0.00 | 0.00 | 0.00 | 0.00 | 0.00 | 0.00 | 0.00 | 4.41E+06 | 3.33E+06 | 0.00 | 0.00 | 0.00 | 0.00 | 0.00 | 0.00 |
| **89** | 8.21 | 0.00 | 0.00 | 0.00 | 0.00 | 0.00 | 0.00 | 0.00 | 0.00 | 0.00 | 0.00 | 0.00 | 0.00 | 0.00 | 0.00 | 0.00 | 0.00 | 7.92E+05 | 0.00 |
| **90** | 15.21 | 0.00 | 0.00 | 0.00 | 0.00 | 2.16E+05 | 0.00 | 0.00 | 0.00 | 0.00 | 8.57E+04 | 0.00 | 0.00 | 0.00 | 0.00 | 0.00 | 0.00 | 0.00 | 0.00 |
| **91** | 4.54 | 0.00 | 0.00 | 0.00 | 0.00 | 0.00 | 0.00 | 0.00 | 0.00 | 0.00 | 8.78E+06 | 0.00 | 0.00 | 0.00 | 0.00 | 0.00 | 0.00 | 0.00 | 0.00 |
| **92** | 8.71 | 0.00 | 0.00 | 0.00 | 0.00 | 0.00 | 0.00 | 0.00 | 0.00 | 0.00 | 0.00 | 0.00 | 1.45 | 0.00 | 0.00 | 0.00 | 0.00 | 0.00 | 0.00 |
| **93** | 3.85 | 0.00 | 0.00 | 0.00 | 0.00 | 5.14E+06 | 0.00 | 0.00 | 0.00 | 0.00 | 0.00 | 0.00 | 0.00 | 0.00 | 0.00 | 0.00 | 1.49E+04 | 0.00 | 2.93E+04 |
| **94** | 25.64 | 0.00 | 0.00 | 2.80E+02 | 0.00 | 0.00 | 0.00 | 0.00 | 0.00 | 0.00 | 0.00 | 0.00 | 0.00 | 0.00 | 0.00 | 0.00 | 0.00 | 0.00 | 0.00 |
| **95** | 13.64 | 0.00 | 0.00 | 0.00 | 1.03E+08 | 0.00 | 0.00 | 0.00 | 0.00 | 1.40E+04 | 0.00 | 0.00 | 3.93E+05 | 1.03E+08 | 0.00 | 0.00 | 0.00 | 0.00 | 0.00 |
| **96** | 69.30 | 0.00 | 0.00 | 0.00 | 0.00 | 0.00 | 0.00 | 0.00 | 0.00 | 0.00 | 1.50E+04 | 0.00 | 0.00 | 0.00 | 0.00 | 0.00 | 0.00 | 0.00 | 4.33E+04 |
| **97** | 27.10 | 0.00 | 0.00 | 8.48E+05 | 0.00 | 0.00 | 0.00 | 0.00 | 0.00 | 0.00 | 0.00 | 0.00 | 0.00 | 0.00 | 0.00 | 0.00 | 0.00 | 0.00 | 0.00 |
| **98** | 22.96 | 7.76E+07 | 0.00 | 1.78E+04 | 0.00 | 0.00 | 0.00 | 0.00 | 0.00 | 0.00 | 0.00 | 0.00 | 0.00 | 0.00 | 2.99E+05 | 1.08E+03 | 0.00 | 0.00 | 1.01E+05 |
| **99** | 24.25 | 0.00 | 0.00 | 1.84E+08 | 0.00 | 4.77E+03 | 0.00 | 0.00 | 0.00 | 0.00 | 0.00 | 0.00 | 0.00 | 0.00 | 0.00 | 0.00 | 0.00 | 2.22E+03 | 1.13E+06 |
| **100** | 7.14 | 0.00 | 0.00 | 0.00 | 0.00 | 0.00 | 0.00 | 0.00 | 0.00 | 0.00 | 0.00 | 0.00 | 0.00 | 0.00 | 0.00 | 0.00 | 0.00 | 0.00 | 0.00 |
| **101** | 13.45 | 0.00 | 0.00 | 0.00 | 1.69E+05 | 1.02E+08 | 0.00 | 0.00 | 3.34E+06 | 0.00 | 0.00 | 0.00 | 0.00 | 0.00 | 0.00 | 0.00 | 1.41E+06 | 0.00 | 0.00 |
| **102** | 7.33 | 0.00 | 0.00 | 0.00 | 0.00 | 0.00 | 0.00 | 0.00 | 0.00 | 0.00 | 0.00 | 0.00 | 0.00 | 0.00 | 0.00 | 0.00 | 0.00 | 0.00 | 0.00 |
| **103** | 27.08 | 0.00 | 0.00 | 0.00 | 0.00 | 1.25E+02 | 2.66E+06 | 0.00 | 0.00 | 0.00 | 0.00 | 0.00 | 8.85E+07 | 0.00 | 0.00 | 0.00 | 0.00 | 0.00 | 2.28E+07 |
| **104** | 11.67 | 0.00 | 0.00 | 2.52E+06 | 0.00 | 0.00 | 0.00 | 0.00 | 0.00 | 0.00 | 0.00 | 0.00 | 0.00 | 0.00 | 0.00 | 0.00 | 0.00 | 0.00 | 0.00 |
| **105** | 49.73 | 0.00 | 0.00 | 0.00 | 0.00 | 0.00 | 4.36E+04 | 0.00 | 0.00 | 0.00 | 0.00 | 0.00 | 5.80E+06 | 0.00 | 0.00 | 7.47E+06 | 0.00 | 0.00 | 0.00 |
| **106** | 20.15 | 0.00 | 0.00 | 7.15E+04 | 0.00 | 0.00 | 0.00 | 0.00 | 0.00 | 0.00 | 2.99E+03 | 0.00 | 0.00 | 0.00 | 0.00 | 0.00 | 0.00 | 0.00 | 0.00 |
| **107** | 29.51 | 0.00 | 0.00 | 0.00 | 0.00 | 0.00 | 0.00 | 0.00 | 0.00 | 0.00 | 5.19E+07 | 0.00 | 0.00 | 0.00 | 0.00 | 0.00 | 0.00 | 0.00 | 0.00 |
| **108** | 33.99 | 0.00 | 0.00 | 0.00 | 0.00 | 0.00 | 0.00 | 0.00 | 0.00 | 4.25E+05 | 0.00 | 0.00 | 0.00 | 0.00 | 0.00 | 0.00 | 0.00 | 0.00 | 0.00 |
| **109** | 27.89 | 0.00 | 0.00 | 0.00 | 0.00 | 0.00 | 0.00 | 0.00 | 0.00 | 0.00 | 1.93E+06 | 0.00 | 0.00 | 0.00 | 0.00 | 0.00 | 0.00 | 0.00 | 2.36E+04 |
| **110** | 1.98 | 0.00 | 0.00 | 0.00 | 0.00 | 0.00 | 0.00 | 0.00 | 0.00 | 0.00 | 0.00 | 0.00 | 0.00 | 0.00 | 0.00 | 0.00 | 0.00 | 0.00 | 0.00 |
| **111** | 85.34 | 0.00 | 0.00 | 1.67E+06 | 0.00 | 0.65 | 0.00 | 0.00 | 0.00 | 0.00 | 0.00 | 0.00 | 0.00 | 0.00 | 0.00 | 0.00 | 0.00 | 0.00 | 0.00 |
| **112** | 7.89 | 0.00 | 0.00 | 0.00 | 0.00 | 0.00 | 0.00 | 0.00 | 0.00 | 0.00 | 0.00 | 0.00 | 0.00 | 0.00 | 1.99E+06 | 0.00 | 0.00 | 0.00 | 0.00 |
| **113** | 79.38 | 0.00 | 0.00 | 0.00 | 0.00 | 0.00 | 0.00 | 0.00 | 0.00 | 0.00 | 0.00 | 0.00 | 0.00 | 0.00 | 0.00 | 0.00 | 0.00 | 0.00 | 0.00 |
| **114** | 2.95 | 0.00 | 0.00 | 0.00 | 0.00 | 0.00 | 0.00 | 0.00 | 0.00 | 0.00 | 0.00 | 0.00 | 0.00 | 0.00 | 0.00 | 0.00 | 0.00 | 0.00 | 0.00 |
| **115** | 23.64 | 0.00 | 0.00 | 0.00 | 0.00 | 0.00 | 0.00 | 7.48E+03 | 0.00 | 0.00 | 3.41E+04 | 3.88E+06 | 0.00 | 0.00 | 0.00 | 0.00 | 0.00 | 0.00 | 0.00 |
| **116** | 11.31 | 0.00 | 0.00 | 0.00 | 0.00 | 0.00 | 0.00 | 0.00 | 0.00 | 0.00 | 0.00 | 0.00 | 4.17E+06 | 0.00 | 0.00 | 0.00 | 0.00 | 0.00 | 0.00 |
| **117** | 1.06 | 0.00 | 0.00 | 0.00 | 0.00 | 1.25E+03 | 0.00 | 0.00 | 0.00 | 0.00 | 0.00 | 2.29E+05 | 0.00 | 0.00 | 0.00 | 0.00 | 0.00 | 0.00 | 0.00 |
| **118** | 59.81 | 0.00 | 0.00 | 0.00 | 0.00 | 0.00 | 0.00 | 0.00 | 0.00 | 0.00 | 3.54E+05 | 0.00 | 0.00 | 0.00 | 0.00 | 0.00 | 1.08E+08 | 0.00 | 0.00 |
| **119** | 7.99 | 0.00 | 0.00 | 0.00 | 0.00 | 0.00 | 0.00 | 0.00 | 0.00 | 0.00 | 0.00 | 0.00 | 0.00 | 0.00 | 0.00 | 0.00 | 0.00 | 0.00 | 0.00 |

**Supplementary Table 3.** Raw human (h) and HPV DNA genotyping outcomes (copies/µl DNA extract) in clinician-collected cervical samples using the Riatol quantitative PCR HPV genotyping assay. NA: not available.

| **ID** | **hDNA**  **(ng/µl)** | **Clinician-collected cervical samples (copies/µl)** | | | | | | | | | | | | | | | | | |
| --- | --- | --- | --- | --- | --- | --- | --- | --- | --- | --- | --- | --- | --- | --- | --- | --- | --- | --- | --- |
|  |  | **HPV6** | **HPV11** | **HPV16** | **HPV18** | **HPV31** | **HPV33** | **HPV35** | **HPV39** | **HPV45** | **HPV51** | **HPV52** | **HPV53** | **HPV56** | **HPV58** | **HPV59** | **HPV66** | **HPV67** | **HPV68** |
| **1** | 29.09 | 0.00 | 0.00 | 0.00 | 0.00 | 0.00 | 0.00 | 0.00 | 0.00 | 0.00 | 0.00 | 0.00 | 0.00 | 0.00 | 0.00 | 0.00 | 0.00 | 0.00 | 0.00 |
| **2** | 37.66 | 0.00 | 0.00 | 0.00 | 0.00 | 0.00 | 0.00 | 2.85E+08 | 0.00 | 0.00 | 0.00 | 0.00 | 0.00 | 0.00 | 0.00 | 0.00 | 0.00 | 2.85E+08 | 0.00 |
| **3** | 25.61 | 0.00 | 0.00 | 0.00 | 0.00 | 5.82E+03 | 0.00 | 0.00 | 0.00 | 0.00 | 0.00 | 0.00 | 0.00 | 0.00 | 0.00 | 0.00 | 0.00 | 0.00 | 0.00 |
| **4** | NA | NA | NA | NA | NA | NA | NA | NA | NA | NA | NA | NA | NA | NA | NA | NA | NA | NA | NA |
| **5** | 43.71 | 0.00 | 0.00 | 1.01E+08 | 2.82E+06 | 0.00 | 0.00 | 0.00 | 0.00 | 0.00 | 0.00 | 0.00 | 0.00 | 0.00 | 0.00 | 0.00 | 3.31E+08 | 0.00 | 3.31E+08 |
| **6** | 21.86 | 0.00 | 0.00 | 0.00 | 0.00 | 0.00 | 0.00 | 0.00 | 0.00 | 0.00 | 0.00 | 0.00 | 0.00 | 0.00 | 0.00 | 0.00 | 0.00 | 0.00 | 0.00 |
| **7** | 2.21E+02 | 0.00 | 0.00 | 0.00 | 0.00 | 0.00 | 0.00 | 0.00 | 0.00 | 0.00 | 0.00 | 0.00 | 0.00 | 0.00 | 0.00 | 0.00 | 0.00 | 0.00 | 0.00 |
| **8** | 40.79 | 0.00 | 0.00 | 0.00 | 0.00 | 0.00 | 0.00 | 0.00 | 0.00 | 0.00 | 0.00 | 0.00 | 0.00 | 0.00 | 0.00 | 0.00 | 0.00 | 0.00 | 0.00 |
| **9** | 3.52E+02 | 0.00 | 0.00 | 0.00 | 0.00 | 0.00 | 0.00 | 0.00 | 0.00 | 0.00 | 0.00 | 0.00 | 0.00 | 0.00 | 0.00 | 0.00 | 0.00 | 0.00 | 0.00 |
| **10** | 38.87 | 0.00 | 0.00 | 0.00 | 0.00 | 0.00 | 0.00 | 0.00 | 0.00 | 0.00 | 0.00 | 0.00 | 0.00 | 0.00 | 0.00 | 3.24 | 0.00 | 0.00 | 0.00 |
| **11** | 2.21E+02 | 0.00 | 0.00 | 0.00 | 0.00 | 0.00 | 0.00 | 0.00 | 0.00 | 0.00 | 0.00 | 0.00 | 0.00 | 0.00 | 0.00 | 0.00 | 2.68E+05 | 0.00 | 0.00 |
| **12** | 35.62 | 0.00 | 0.00 | 0.00 | 0.00 | 2.50E+03 | 2.70E+08 | 0.00 | 72.86 | 0.00 | 0.00 | 0.00 | 0.00 | 0.00 | 2.70E+08 | 0.00 | 0.00 | 0.00 | 0.00 |
| **13** | 91.69 | 0.00 | 0.00 | 0.00 | 0.00 | 0.00 | 0.00 | 0.00 | 0.00 | 0.00 | 0.00 | 0.00 | 0.00 | 0.00 | 0.00 | 0.00 | 0.00 | 0.00 | 0.00 |
| **14** | 27.98 | 0.00 | 0.00 | 0.00 | 0.00 | 0.00 | 0.00 | 0.00 | 0.00 | 0.00 | 0.00 | 0.00 | 0.00 | 0.00 | 0.00 | 0.00 | 0.00 | 0.00 | 0.00 |
| **15** | 1.19E+02 | 0.00 | 0.00 | 0.00 | 0.00 | 4.52E+04 | 0.00 | 0.00 | 0.00 | 0.00 | 0.00 | 0.00 | 0.00 | 0.00 | 0.00 | 0.00 | 0.00 | 0.00 | 0.00 |
| **16** | 74.53 | 0.00 | 0.00 | 3.95E+04 | 0.00 | 0.00 | 0.00 | 3.50E+05 | 0.00 | 0.00 | 0.00 | 2.02E+03 | 0.00 | 0.00 | 0.00 | 0.00 | 0.00 | 0.00 | 0.00 |
| **17** | 67.08 | 0.00 | 0.00 | 0.00 | 0.00 | 0.00 | 0.00 | 0.00 | 0.00 | 0.00 | 0.00 | 0.00 | 0.00 | 0.00 | 0.00 | 0.00 | 0.00 | 0.00 | 0.00 |
| **18** | 1.10E+02 | 0.00 | 0.00 | 0.00 | 0.00 | 0.00 | 0.00 | 0.00 | 4.27E+02 | 0.00 | 0.00 | 0.00 | 0.00 | 0.00 | 0.00 | 3.33 | 0.00 | 2.06E+02 | 0.00 |
| **19** | 1.63E+02 | 0.00 | 0.00 | 0.00 | 0.00 | 0.00 | 0.00 | 0.00 | 0.00 | 0.00 | 0.00 | 0.00 | 0.00 | 0.00 | 0.00 | 0.00 | 0.00 | 0.00 | 0.00 |
| **20** | 53.90 | 0.00 | 0.00 | 0.00 | 0.00 | 0.00 | 0.00 | 0.00 | 0.00 | 0.00 | 0.00 | 0.00 | 0.00 | 0.00 | 0.00 | 0.00 | 0.00 | 0.00 | 0.00 |
| **21** | 60.67 | 0.00 | 0.00 | 0.00 | 0.00 | 4.60E+08 | 0.00 | 0.00 | 0.00 | 0.00 | 0.00 | 0.00 | 0.00 | 0.00 | 0.00 | 0.00 | 0.00 | 0.00 | 0.00 |
| **22** | 2.53E+02 | 0.00 | 0.00 | 0.00 | 0.00 | 0.00 | 0.00 | 0.00 | 0.00 | 0.00 | 0.00 | 0.00 | 0.00 | 0.00 | 0.00 | 0.00 | 0.00 | 0.00 | 0.00 |
| **23** | 43.99 | 1.28E+06 | 4.76E+06 | 6.36E+06 | 0.00 | 0.00 | 0.00 | 0.00 | 1.00E+04 | 0.00 | 0.00 | 0.00 | 1.67E+04 | 0.00 | 0.00 | 0.00 | 0.00 | 0.00 | 0.00 |
| **24** | 75.22 | 0.00 | 0.00 | 0.00 | 0.00 | 0.00 | 0.00 | 0.00 | 3.79E+03 | 0.00 | 0.00 | 0.00 | 0.00 | 0.00 | 0.00 | 0.00 | 0.00 | 0.00 | 0.00 |
| **25** | 2.48E+02 | 0.00 | 0.00 | 0.00 | 0.00 | 0.00 | 0.00 | 0.00 | 0.00 | 0.00 | 0.00 | 0.00 | 0.00 | 0.00 | 0.00 | 0.00 | 6.31E+06 | 0.00 | 0.00 |
| **26** | 41.25 | 0.00 | 0.00 | 0.00 | 0.00 | 0.00 | 0.00 | 0.00 | 0.00 | 0.00 | 1.00E+05 | 0.00 | 0.00 | 0.00 | 0.00 | 8.11E+07 | 5.61E+07 | 0.00 | 0.00 |
| **27** | 3.10 | 0.00 | 0.00 | 0.00 | 0.00 | 0.00 | 0.00 | 0.00 | 0.00 | 0.00 | 0.00 | 0.00 | 0.00 | 0.00 | 0.00 | 0.00 | 0.00 | 0.00 | 0.00 |
| **28** | 84.15 | 0.00 | 0.00 | 0.00 | 0.00 | 0.00 | 0.00 | 0.00 | 0.00 | 0.00 | 0.00 | 3.83E+05 | 0.00 | 0.00 | 0.00 | 0.00 | 0.00 | 0.00 | 0.00 |
| **29** | 92.06 | 0.00 | 0.00 | 0.00 | 0.00 | 0.00 | 0.00 | 0.00 | 0.00 | 0.00 | 0.00 | 0.00 | 0.00 | 0.00 | 0.00 | 0.00 | 0.00 | 0.00 | 0.00 |
| **30** | 1.49E+02 | 0.00 | 0.00 | 0.00 | 0.00 | 0.00 | 0.00 | 0.00 | 0.00 | 0.00 | 0.00 | 0.00 | 0.00 | 0.00 | 3.75E+07 | 0.00 | 0.00 | 0.00 | 0.00 |
| **31** | 2.37E+02 | 0.00 | 0.00 | 0.00 | 0.00 | 0.00 | 3.00E+08 | 0.00 | 0.00 | 0.00 | 0.00 | 0.00 | 0.00 | 0.00 | 0.00 | 0.00 | 0.00 | 0.00 | 0.00 |
| **32** | 47.32 | 0.00 | 0.00 | 0.00 | 0.00 | 1.79E+04 | 0.00 | 0.00 | 0.00 | 0.00 | 0.00 | 0.00 | 0.00 | 0.00 | 0.00 | 0.00 | 0.00 | 0.00 | 0.00 |
| **33** | NA | NA | NA | NA | NA | NA | NA | NA | NA | NA | NA | NA | NA | NA | NA | NA | NA | NA | NA |
| **34** | 2.89E+02 | 0.00 | 0.00 | 0.00 | 0.00 | 0.00 | 0.00 | 0.00 | 0.00 | 0.00 | 0.00 | 0.00 | 0.00 | 0.00 | 0.00 | 0.00 | 0.00 | 0.00 | 0.00 |
| **35** | 79.86 | 2.26E+08 | 0.00 | 0.00 | 0.00 | 0.00 | 0.00 | 0.00 | 0.00 | 0.00 | 0.00 | 0.00 | 0.00 | 0.00 | 0.00 | 1.22E+07 | 1.35E+06 | 0.00 | 0.00 |
| **36** | 44.78 | 0.00 | 0.00 | 0.00 | 0.00 | 1.07E+07 | 0.00 | 0.00 | 0.00 | 0.00 | 0.00 | 0.00 | 0.00 | 0.00 | 0.00 | 0.00 | 0.00 | 0.00 | 0.00 |
| **37** | 70.31 | 0.00 | 0.00 | 1.56E+06 | 0.00 | 0.00 | 0.00 | 0.00 | 0.00 | 0.00 | 0.00 | 0.00 | 0.00 | 0.00 | 0.00 | 0.00 | 0.00 | 0.00 | 0.00 |
| **38** | 3.28E+02 | 0.00 | 0.00 | 2.98E+05 | 0.00 | 0.00 | 0.00 | 0.00 | 0.00 | 0.00 | 0.00 | 0.00 | 0.00 | 0.00 | 0.00 | 0.00 | 0.00 | 0.00 | 0.00 |
| **39** | 33.03 | 0.00 | 0.00 | 0.00 | 0.00 | 0.00 | 0.00 | 0.00 | 0.00 | 0.00 | 2.23E+08 | 0.00 | 0.00 | 0.00 | 0.00 | 0.00 | 8.28E+05 | 0.00 | 0.00 |
| **40** | 1.29E+02 | 0.00 | 0.00 | 0.00 | 0.00 | 8.10E+05 | 0.00 | 0.00 | 0.00 | 0.00 | 0.00 | 0.00 | 0.00 | 0.00 | 0.00 | 0.00 | 0.00 | 0.00 | 0.00 |
| **41** | NA | NA | NA | NA | NA | NA | NA | NA | NA | NA | NA | NA | NA | NA | NA | NA | NA | NA | NA |
| **42** | 1.27E+02 | 0.00 | 0.00 | 0.00 | 0.00 | 0.00 | 0.00 | 0.00 | 0.00 | 0.00 | 0.00 | 0.00 | 0.00 | 0.00 | 0.00 | 0.00 | 0.00 | 0.00 | 0.00 |
| **43** | 44.13 | 0.00 | 0.00 | 0.00 | 0.00 | 0.00 | 0.00 | 0.00 | 0.00 | 0.00 | 0.00 | 0.00 | 0.00 | 0.00 | 0.00 | 0.00 | 0.00 | 0.00 | 0.00 |
| **44** | 48.31 | 0.00 | 0.00 | 0.00 | 0.00 | 0.00 | 0.00 | 0.00 | 0.00 | 0.00 | 1.49E+06 | 0.00 | 0.00 | 0.00 | 0.00 | 0.00 | 0.00 | 0.00 | 0.00 |
| **45** | NA | NA | NA | NA | NA | NA | NA | NA | NA | NA | NA | NA | NA | NA | NA | NA | NA | NA | NA |
| **46** | 1.17E+02 | 0.00 | 26595.45 | 8.87E+03 | 0.00 | 0.00 | 0.00 | 0.00 | 0.00 | 5.38E+08 | 0.00 | 0.00 | 0.00 | 0.00 | 0.00 | 0.00 | 0.00 | 0.00 | 1.02E+06 |
| **47** | 1.34E+02 | 0.00 | 0.00 | 0.00 | 0.00 | 0.00 | 0.00 | 0.00 | 0.00 | 0.00 | 0.00 | 0.00 | 0.00 | 0.00 | 0.00 | 0.00 | 0.00 | 0.00 | 0.00 |
| **48** | 95.46 | 0.00 | 0.00 | 2.31E+05 | 0.00 | 0.00 | 0.00 | 0.00 | 0.00 | 0.00 | 0.00 | 2.39E+06 | 0.00 | 0.00 | 0.00 | 0.00 | 0.00 | 0.00 | 0.00 |
| **49** | 1.64E+02 | 4.15E+07 | 0.00 | 0.00 | 0.00 | 1.24E+04 | 0.00 | 1.24E+09 | 0.00 | 0.00 | 0.00 | 0.00 | 0.00 | 6.02E+07 | 0.00 | 0.00 | 0.00 | 0.00 | 0.00 |
| **50** | 38.50 | 0.00 | 0.00 | 0.00 | 0.00 | 0.00 | 0.00 | 0.00 | 0.00 | 0.00 | 0.00 | 0.00 | 0.00 | 0.00 | 0.00 | 0.00 | 1.61E+07 | 0.00 | 0.00 |
| **51** | 1.04E+02 | 0.00 | 0.00 | 0.00 | 0.00 | 0.00 | 0.00 | 0.00 | 0.00 | 3.16E+04 | 0.00 | 0.00 | 9.18E+07 | 0.00 | 0.00 | 0.00 | 0.00 | 1.26E+07 | 0.00 |
| **52** | 16.20 | 0.00 | 0.00 | 0.00 | 0.00 | 0.00 | 0.00 | 0.00 | 0.00 | 0.00 | 0.00 | 0.00 | 0.00 | 8.08E+05 | 0.00 | 0.00 | 0.00 | 0.00 | 0.00 |
| **53** | 1.77E+02 | 0.00 | 0.00 | 4.64E+07 | 0.00 | 0.00 | 0.00 | 0.00 | 0.00 | 0.00 | 0.00 | 0.00 | 0.00 | 0.00 | 0.00 | 0.00 | 0.00 | 0.00 | 0.00 |
| **54** | 11.53 | 0.00 | 0.00 | 0.00 | 0.00 | 0.00 | 0.00 | 0.00 | 8.73E+07 | 0.00 | 0.00 | 0.00 | 7.77E+07 | 0.00 | 0.00 | 0.00 | 0.00 | 0.00 | 0.00 |
| **55** | 30.68 | 0.00 | 0.00 | 8.83E+07 | 0.00 | 0.00 | 0.00 | 0.00 | 0.00 | 0.00 | 0.00 | 0.00 | 0.00 | 0.00 | 0.00 | 0.00 | 0.00 | 0.00 | 0.00 |
| **56** | 62.87 | 0.00 | 0.00 | 0.00 | 0.00 | 0.00 | 0.00 | 0.00 | 0.00 | 0.00 | 0.00 | 0.00 | 0.00 | 0.00 | 0.00 | 0.00 | 0.00 | 0.00 | 0.00 |
| **57** | 30.02 | 0.00 | 0.00 | 0.00 | 0.00 | 2.27E+08 | 0.00 | 0.00 | 0.00 | 0.00 | 0.00 | 0.00 | 0.00 | 0.00 | 0.00 | 0.00 | 0.00 | 0.00 | 0.00 |
| **58** | 76.29 | 0.00 | 0.00 | 2.05E+06 | 0.00 | 0.00 | 0.00 | 0.00 | 0.00 | 0.00 | 5.46E+06 | 0.00 | 0.00 | 0.00 | 0.00 | 0.00 | 0.00 | 0.00 | 0.00 |
| **59** | 1.05E+02 | 2.80E+08 | 0.00 | 0.00 | 0.00 | 0.00 | 0.00 | 0.00 | 0.00 | 0.00 | 0.00 | 0.00 | 6.11E+06 | 0.00 | 0.00 | 0.00 | 0.00 | 0.00 | 0.00 |
| **60** | 54.21 | 0.00 | 0.00 | 3.23E+08 | 0.00 | 0.00 | 0.00 | 0.00 | 0.00 | 0.00 | 0.00 | 0.00 | 0.00 | 0.00 | 0.00 | 0.00 | 0.00 | 0.00 | 0.00 |
| **61** | 2.45E+02 | 0.00 | 0.00 | 8.66E+06 | 0.00 | 0.00 | 0.00 | 0.00 | 0.00 | 0.00 | 0.00 | 1.43E+06 | 0.00 | 0.00 | 0.00 | 0.00 | 0.00 | 0.00 | 0.00 |
| **62** | 10.20 | 0.00 | 0.00 | 0.00 | 0.00 | 7.73E+07 | 0.00 | 0.00 | 0.00 | 0.00 | 0.00 | 0.00 | 0.00 | 0.00 | 0.00 | 0.00 | 0.00 | 0.00 | 3.16E+06 |
| **63** | 5.43 | 0.00 | 0.00 | 0.00 | 6.21E+04 | 0.00 | 0.00 | 0.00 | 0.00 | 0.00 | 0.00 | 0.00 | 0.00 | 0.00 | 0.00 | 0.00 | 0.00 | 0.00 | 0.00 |
| **64** | NA | 0.00 | 0.00 | 0.00 | 0.00 | 1.29E+07 | 0.00 | 0.00 | 0.00 | 0.00 | 0.00 | 0.00 | 0.00 | 0.00 | 0.00 | 0.00 | 0.00 | 0.00 | 0.00 |
| **65** | 71.47 | 0.00 | 0.00 | 0.00 | 9.26E+05 | 0.00 | 0.00 | 0.00 | 0.00 | 0.00 | 0.00 | 0.00 | 0.00 | 0.00 | 0.00 | 0.00 | 0.00 | 0.00 | 0.00 |
| **66** | 1.24E+02 | 0.00 | 0.00 | 0.00 | 1.20E+06 | 0.00 | 0.00 | 0.00 | 0.00 | 0.00 | 0.00 | 0.00 | 0.00 | 0.00 | 0.00 | 0.00 | 0.00 | 0.00 | 0.00 |
| **67** | 17.79 | 0.00 | 0.00 | 0.00 | 0.00 | 1.20E+06 | 0.00 | 0.00 | 0.00 | 0.00 | 0.00 | 0.00 | 0.00 | 0.00 | 0.00 | 0.00 | 0.00 | 0.00 | 0.00 |
| **68** | 1.09E+02 | 0.00 | 0.00 | 0.00 | 0.00 | 0.00 | 0.00 | 0.00 | 0.00 | 0.00 | 0.00 | 0.00 | 0.00 | 0.00 | 0.00 | 0.00 | 8.28E+08 | 0.00 | 0.00 |
| **69** | 68.77 | 0.00 | 0.00 | 1.91E+07 | 0.00 | 0.00 | 0.00 | 0.00 | 0.00 | 1.88E+08 | 0.00 | 0.00 | 0.00 | 0.00 | 0.00 | 0.00 | 0.00 | 0.00 | 0.00 |
| **70** | 1.75E+02 | 0.00 | 0.00 | 0.00 | 0.00 | 4.88E+07 | 0.00 | 0.00 | 0.00 | 0.00 | 0.00 | 0.00 | 0.00 | 0.00 | 0.00 | 0.00 | 0.00 | 0.00 | 0.00 |
| **71** | 1.17E+02 | 0.00 | 0.00 | 8.86E+03 | 0.00 | 0.00 | 0.00 | 0.00 | 0.00 | 0.00 | 0.00 | 0.00 | 0.00 | 0.00 | 0.00 | 0.00 | 0.00 | 0.00 | 0.00 |
| **72** | 5.20 | 0.00 | 0.00 | 0.00 | 0.00 | 0.00 | 0.00 | 0.00 | 0.00 | 0.00 | 1.42E+05 | 0.00 | 0.00 | 0.00 | 0.00 | 0.00 | 0.00 | 0.00 | 5.14E+05 |
| **73** | 77.64 | 0.00 | 0.00 | 0.00 | 0.00 | 1.03E+07 | 0.00 | 0.00 | 0.00 | 0.00 | 0.00 | 0.00 | 1.77E+06 | 0.00 | 0.00 | 0.00 | 0.00 | 0.00 | 0.00 |
| **74** | 52.78 | 0.00 | 0.00 | 0.00 | 0.00 | 0.00 | 0.00 | 0.00 | 0.00 | 0.00 | 1.60E+06 | 0.00 | 0.00 | 0.00 | 0.00 | 0.00 | 0.00 | 0.00 | 0.00 |
| **75** | 1.00E+02 | 0.00 | 0.00 | 0.00 | 0.00 | 0.00 | 0.00 | 0.00 | 0.00 | 0.00 | 0.00 | 0.00 | 0.00 | 0.00 | 0.00 | 0.00 | 0.00 | 0.00 | 0.00 |
| **76** | 1.62E+02 | 0.00 | 0.00 | 0.00 | 0.00 | 0.00 | 0.00 | 0.00 | 0.00 | 0.00 | 0.00 | 0.00 | 0.00 | 0.00 | 0.00 | 0.00 | 0.00 | 0.00 | 1.70E+06 |
| **77** | 1.06E+02 | 0.00 | 0.00 | 2.37E+06 | 0.00 | 0.00 | 0.00 | 0.00 | 8.02E+04 | 0.00 | 0.00 | 0.00 | 0.00 | 0.00 | 0.00 | 0.00 | 0.00 | 0.00 | 0.00 |
| **78** | 3.40 | 0.00 | 0.00 | 0.00 | 0.00 | 2.58E+07 | 0.00 | 0.00 | 0.00 | 0.00 | 0.00 | 0.00 | 0.00 | 0.00 | 0.00 | 0.00 | 0.00 | 0.00 | 0.00 |
| **79** | 15.65 | 0.00 | 0.00 | 0.00 | 0.00 | 0.00 | 0.00 | 0.00 | 0.00 | 0.00 | 0.00 | 0.00 | 0.00 | 0.00 | 0.00 | 0.00 | 0.00 | 0.00 | 0.00 |
| **80** | 48.38 | 0.00 | 0.00 | 0.00 | 0.00 | 0.00 | 0.00 | 0.00 | 0.00 | 0.00 | 0.00 | 5.45E+06 | 0.00 | 1.47E+04 | 0.00 | 0.00 | 0.00 | 0.00 | 0.00 |
| **81** | 1.04E+02 | 0.00 | 0.00 | 2.34E+08 | 0.00 | 0.00 | 0.00 | 3.31E+06 | 0.00 | 0.00 | 1.12E+06 | 0.00 | 0.00 | 4.11E+06 | 0.00 | 0.00 | 3.85E+07 | 0.00 | 0.00 |
| **82** | NA | NA | NA | NA | NA | NA | NA | NA | NA | NA | NA | NA | NA | NA | NA | NA | NA | NA | NA |
| **83** | 1.92E+02 | 0.00 | 0.00 | 9.90E+06 | 0.00 | 0.00 | 0.00 | 0.00 | 0.00 | 0.00 | 0.00 | 0.00 | 0.00 | 0.00 | 0.00 | 0.00 | 0.00 | 0.00 | 1.51E+06 |
| **84** | 6.55 | 0.00 | 0.00 | 0.00 | 0.00 | 0.00 | 0.00 | 0.00 | 0.00 | 0.00 | 0.00 | 0.00 | 4.96E+07 | 0.00 | 0.00 | 0.00 | 0.00 | 0.00 | 0.00 |
| **85** | 83.87 | 0.00 | 0.00 | 0.00 | 0.00 | 2.48E+07 | 0.00 | 0.00 | 0.00 | 0.00 | 0.00 | 0.00 | 0.00 | 0.00 | 0.00 | 0.00 | 0.00 | 0.00 | 0.00 |
| **86** | 1.09E+02 | 0.00 | 0.00 | 0.00 | 0.00 | 8.25E+08 | 0.00 | 5.12E+05 | 0.00 | 0.00 | 0.00 | 0.00 | 0.00 | 0.00 | 0.00 | 0.00 | 0.00 | 0.00 | 0.00 |
| **87** | 1.89E+02 | 0.00 | 0.00 | 0.00 | 0.00 | 0.00 | 0.00 | 0.00 | 0.00 | 0.00 | 0.00 | 5.46E+06 | 0.00 | 0.00 | 0.00 | 2.19E+07 | 0.00 | 0.00 | 1.51E+07 |
| **88** | 15.08 | 0.00 | 0.00 | 0.00 | 0.00 | 0.00 | 0.00 | 0.00 | 0.00 | 0.00 | 0.00 | 3.88E+04 | 7.02E+06 | 0.00 | 0.00 | 0.00 | 0.00 | 0.00 | 0.00 |
| **89** | 41.12 | 0.00 | 0.00 | 0.00 | 0.00 | 0.00 | 0.00 | 0.00 | 0.00 | 0.00 | 0.00 | 0.00 | 0.00 | 0.00 | 0.00 | 0.00 | 0.00 | 1.10E+07 | 0.00 |
| **90** | 81.39 | 0.00 | 0.00 | 0.00 | 0.00 | 3.43E+07 | 0.00 | 0.00 | 0.00 | 0.00 | 1.48E+06 | 0.00 | 0.00 | 0.00 | 0.00 | 0.00 | 0.00 | 0.00 | 0.00 |
| **91** | 68.49 | 0.00 | 0.00 | 0.00 | 0.00 | 0.00 | 4.52E+06 | 0.00 | 0.00 | 0.00 | 3.34E+07 | 0.00 | 0.00 | 0.00 | 0.00 | 0.00 | 0.00 | 0.00 | 0.00 |
| **92** | 31.03 | 0.00 | 0.00 | 0.00 | 0.00 | 0.00 | 0.00 | 0.00 | 0.00 | 0.00 | 0.00 | 2.35E+08 | 0.00 | 0.00 | 0.00 | 0.00 | 0.00 | 0.00 | 0.00 |
| **93** | 60.19 | 0.00 | 0.00 | 0.00 | 0.00 | 1.10E+08 | 0.00 | 0.00 | 0.00 | 0.00 | 0.00 | 0.00 | 0.00 | 0.00 | 0.00 | 0.00 | 0.00 | 0.00 | 0.00 |
| **94** | 3.01 | 0.00 | 0.00 | 0.00 | 0.00 | 0.00 | 0.00 | 0.00 | 0.00 | 0.00 | 0.00 | 0.00 | 0.00 | 0.00 | 0.00 | 0.00 | 0.00 | 0.00 | 0.00 |
| **95** | 19.72 | 0.00 | 0.00 | 0.00 | 8.46E+07 | 0.00 | 0.00 | 0.00 | 0.00 | 0.00 | 0.00 | 0.00 | 9.59E+05 | 1.49E+08 | 0.00 | 0.00 | 0.00 | 0.00 | 0.00 |
| **96** | 2.00E+02 | 0.00 | 0.00 | 0.00 | 0.00 | 0.00 | 0.00 | 0.00 | 0.00 | 0.00 | 4.54E+04 | 0.00 | 0.00 | 4.54E+04 | 0.00 | 0.00 | 0.00 | 0.00 | 1.06E+05 |
| **97** | 73.75 | 0.00 | 0.00 | 5.18E+07 | 0.00 | 0.00 | 0.00 | 0.00 | 0.00 | 0.00 | 0.00 | 0.00 | 0.00 | 0.00 | 0.00 | 0.00 | 0.00 | 0.00 | 0.00 |
| **98** | 50.32 | 0.00 | 0.00 | 2.34E+07 | 0.00 | 0.00 | 0.00 | 0.00 | 0.00 | 0.00 | 0.00 | 0.00 | 0.00 | 0.00 | 0.00 | 0.00 | 0.00 | 0.00 | 0.00 |
| **99** | 41.73 | 0.00 | 0.00 | 3.16E+08 | 0.00 | 12.01 | 0.00 | 0.00 | 0.00 | 0.00 | 0.00 | 0.00 | 0.00 | 0.00 | 0.00 | 0.00 | 0.00 | 0.00 | 5.29E+06 |
| **100** | 28.30 | 0.00 | 0.00 | 0.00 | 0.00 | 0.00 | 0.00 | 0.00 | 0.00 | 0.00 | 0.00 | 0.00 | 0.00 | 0.00 | 0.00 | 0.00 | 0.00 | 0.00 | 0.00 |
| **101** | 35.59 | 0.00 | 0.00 | 0.00 | 4.48E+05 | 2.70E+08 | 0.00 | 0.00 | 4.72E+07 | 0.00 | 0.00 | 0.00 | 0.00 | 0.00 | 0.00 | 0.00 | 0.00 | 0.00 | 0.00 |
| **102** | 98.13 | 0.00 | 0.00 | 0.00 | 0.00 | 0.00 | 0.00 | 0.00 | 0.00 | 0.00 | 0.00 | 0.00 | 0.00 | 0.00 | 0.00 | 0.00 | 0.00 | 0.00 | 0.00 |
| **103** | 28.16 | 0.00 | 0.00 | 0.00 | 0.00 | 0.00 | 1.92E+07 | 0.00 | 0.00 | 0.00 | 0.00 | 0.00 | 7.51E+05 | 0.00 | 0.00 | 0.00 | 0.00 | 0.00 | 5.27E+07 |
| **104** | 37.08 | 0.00 | 0.00 | 2.81E+08 | 0.00 | 0.00 | 0.00 | 0.00 | 0.00 | 0.00 | 0.00 | 0.00 | 0.00 | 0.00 | 0.00 | 0.00 | 0.00 | 0.00 | 0.00 |
| **105** | 1.57E+02 | 0.00 | 0.00 | 0.00 | 0.00 | 0.00 | 0.00 | 0.00 | 0.00 | 0.00 | 0.00 | 0.00 | 0.00 | 0.00 | 0.00 | 1.59E+08 | 0.00 | 0.00 | 0.00 |
| **106** | 21.26 | 0.00 | 0.00 | 0.00 | 0.00 | 0.00 | 0.00 | 0.00 | 0.00 | 0.00 | 0.00 | 0.00 | 0.00 | 0.00 | 0.00 | 0.00 | 0.00 | 0.00 | 0.00 |
| **107** | 27.45 | 0.00 | 0.00 | 0.00 | 0.00 | 0.00 | 0.00 | 0.00 | 0.00 | 0.00 | 2.08E+08 | 0.00 | 0.00 | 0.00 | 0.00 | 0.00 | 0.00 | 0.00 | 0.00 |
| **108** | 26.14 | 0.00 | 0.00 | 0.00 | 0.00 | 0.00 | 0.00 | 0.00 | 0.00 | 9.72E+05 | 0.00 | 0.00 | 0.00 | 0.00 | 0.00 | 0.00 | 0.00 | 0.00 | 0.00 |
| **109** | 72.59 | 0.00 | 0.00 | 0.00 | 0.00 | 0.00 | 0.00 | 0.00 | 0.00 | 0.00 | 4.34E+06 | 0.00 | 0.00 | 0.00 | 0.00 | 0.00 | 0.00 | 0.00 | 0.00 |
| **110** | 13.75 | 0.00 | 0.00 | 0.00 | 0.00 | 0.00 | 0.00 | 0.00 | 0.00 | 0.00 | 0.00 | 0.00 | 0.00 | 0.00 | 0.00 | 0.00 | 0.00 | 0.00 | 0.00 |
| **111** | 20.66 | 0.00 | 0.00 | 1.66E+06 | 0.00 | 0.00 | 0.00 | 0.00 | 0.00 | 0.00 | 0.00 | 0.00 | 0.00 | 0.00 | 0.00 | 0.00 | 0.00 | 0.00 | 0.00 |
| **112** | 1.13E+02 | 0.00 | 0.00 | 0.00 | 0.00 | 0.00 | 0.00 | 0.00 | 0.00 | 0.00 | 0.00 | 1.28E+05 | 0.00 | 0.00 | 2.08E+08 | 0.00 | 0.00 | 0.00 | 0.00 |
| **113** | 38.17 | 0.00 | 0.00 | 0.00 | 0.00 | 0.00 | 0.00 | 0.00 | 0.00 | 0.00 | 0.00 | 0.00 | 0.00 | 0.00 | 0.00 | 0.00 | 0.00 | 0.00 | 0.00 |
| **114** | 1.37E+02 | 0.00 | 0.00 | 0.00 | 0.00 | 0.00 | 0.00 | 0.00 | 0.00 | 0.00 | 0.00 | 0.00 | 0.00 | 0.00 | 0.00 | 0.00 | 0.00 | 0.00 | 0.00 |
| **115** | 39.43 | 0.00 | 0.00 | 0.00 | 0.00 | 0.00 | 0.00 | 1.79E+05 | 0.00 | 0.00 | 0.00 | 1.59E+08 | 0.00 | 0.00 | 0.00 | 0.00 | 0.00 | 0.00 | 0.00 |
| **116** | 1.51E+02 | 0.00 | 0.00 | 0.00 | 0.00 | 0.00 | 0.00 | 0.00 | 0.00 | 0.00 | 0.00 | 0.00 | 3.61E+07 | 0.00 | 0.00 | 0.00 | 0.00 | 0.00 | 0.00 |
| **117** | 38.01 | 0.00 | 0.00 | 0.00 | 0.00 | 1.08E+07 | 0.00 | 0.00 | 0.00 | 0.00 | 0.00 | 2.10E+08 | 0.00 | 0.00 | 0.00 | 0.00 | 0.00 | 0.00 | 0.00 |
| **118** | 2.34E+02 | 0.00 | 0.00 | 0.00 | 0.00 | 0.00 | 0.00 | 0.00 | 0.00 | 0.00 | 0.00 | 0.00 | 0.00 | 0.00 | 0.00 | 0.00 | 1.40E+09 | 0.00 | 0.00 |
| **119** | 1.91E+02 | 0.00 | 0.00 | 0.00 | 0.00 | 0.00 | 4.83E+06 | 0.00 | 0.00 | 0.00 | 0.00 | 0.00 | 0.00 | 0.00 | 0.00 | 0.00 | 0.00 | 0.00 | 0.00 |

**Supplementary Fig. 1.** Evaluation of triage marker performance in first-void urine to discern high-grade disease using the area under the curve (AUC). Performance of each marker was assessed using cytology ((A) HSIL+, n=89) and histology ((B) CIN2+ and (C) CIN3, n=33) as reference. AUC’s for each marker are visualized by receiver operating characteristic-curves (95% confidence intervals are detailed in **Table 1**).

**Reference**

1 IARC. Agents classified by the IARC Monographs. (2016).
